# A mixture of mobility and meteorological data provides a high correlation with COVID-19 growth in an infection-naive population: a study for Spanish provinces

**DOI:** 10.3389/fpubh.2024.1288531

**Published:** 2024-03-07

**Authors:** David Conesa, Víctor López de Rioja, Tania Gullón, Adriá Tauste Campo, Clara Prats, Enrique Alvarez-Lacalle, Blas Echebarria

**Affiliations:** ^1^Department of Physics, Universitat Politécnica de Catalunya, Barcelona, Spain; ^2^Spanish Ministry of Transport, Mobility and Urban Agenda (MITMA), Madrid, Spain

**Keywords:** epidemiology, COVID-19, mobility, meteorological data, statistical correlations

## Abstract

**Introduction:**

We use Spanish data from August 2020 to March 2021 as a natural experiment to analyze how a standardized measure of COVID-19 growth correlates with asymmetric meteorological and mobility situations in 48 Spanish provinces. The period of time is selected prior to vaccination so that the level of susceptibility was high, and during geographically asymmetric implementation of non-pharmacological interventions.

**Methods:**

We develop reliable aggregated mobility data from different public sources and also compute the average meteorological time series of temperature, dew point, and UV radiance in each Spanish province from satellite data. We perform a dimensionality reduction of the data using principal component analysis and investigate univariate and multivariate correlations of mobility and meteorological data with COVID-19 growth.

**Results:**

We find significant, but generally weak, univariate correlations for weekday aggregated mobility in some, but not all, provinces. On the other hand, principal component analysis shows that the different mobility time series can be properly reduced to three time series. A multivariate time-lagged canonical correlation analysis of the COVID-19 growth rate with these three time series reveals a highly significant correlation, with a median R-squared of 0.65. The univariate correlation between meteorological data and COVID-19 growth is generally not significant, but adding its two main principal components to the mobility multivariate analysis increases correlations significantly, reaching correlation coefficients between 0.6 and 0.98 in all provinces with a median R-squared of 0.85. This result is robust to different approaches in the reduction of dimensionality of the data series.

**Discussion:**

Our results suggest an important effect of mobility on COVID-19 cases growth rate. This effect is generally not observed for meteorological variables, although in some Spanish provinces it can become relevant. The correlation between mobility and growth rate is maximal at a time delay of 2-3 weeks, which agrees well with the expected 5?10 day delays between infection, development of symptoms, and the detection/report of the case.

## 1 Introduction

Following the onset of the COVID-19 pandemic in March 2020, measures were taken to avoid or limit transmission. Prior to the development of vaccines, non-pharmaceutical interventions (NPIs) were put in place. Being the virus causing COVID-19, SARS-CoV-2, a virus that is transmitted by the air, some of the first measures implemented were meant to avoid interpersonal contact. Such measures included strict lockdowns, and later on curfews and limits on mobility. In effect, mobility, usually measured by tracking mobile phone positions, was considered a proxy of social interactions, and therefore, was deemed an important advanced indicator of the evolution of the disease.

During the initial stages of the pandemic, within a quasi-naive population, mobility was shown to be correlated with the effective reproduction number, *R*_*t*_, finding that a 10 percentage point reduction in mobility was associated with a 0.04–0.07 reduction in *R*_*t*_ (1). Mobility reductions of about 20–40% were thought to be needed to achieve an *R*_*t*_ below 1.0 (2). Also, a shift in mobility was shown to present a high correlation with death rates one month later (3). Furthermore, within mobility, that related to retail, recreation, and workplaces showed the highest correlation with deaths (4), as well as grocery and pharmacy, and public transport (5). Many other works, at both local and national levels, showed a similar relation between mobility variations and changes in the growth rate of the epidemics, or in the number of deaths (6). In fact, mobility data has been used to improve the prediction of COVID-19 evolution (7). However, some works seem to suggest that, after the first months after March 2020, mobility did not play such an important role in the prediction of transmission, due to the implementation of other non-pharmaceutical interventions (NPIs), as wearing masks, ventilation, etc. (8–10). This could also be related to the existence of super-spreading events, such that focused limitations on the maximum occupations at certain events, could be more effective than overall mobility reduction in hampering transmission (11).

It has also been suggested that seasonal changes affect the transmission, similar to other respiratory diseases (12, 13). Changes in temperature, humidity, and/or UV-radiation have been observed to affect viral transmission (12, 14–17). In the case of temperature, most studies show a negative correlation with growth rate (12, 13), but there are also opposite observations (18). This contradictory data is probably due to the fact that temperature alone cannot explain the changes in disease transmission. A prominent role has also been attributed to UV radiation, which has been shown to decrease both COVID-19 growth rate (19) and associated deaths (20, 21). For the specific case of Spain, a similar negative (but small) correlation has been found between temperature and UV index and COVID-19 incidence and severity (16, 22–24), although, for the first months of the epidemics, no consistent evidence was found regarding the existence of a relationship between the accumulated number of COVID-19 cases and temperature values at the province level (25). Studies of the combined effect of seasonal environmental factors and human mobility (26), show that UV-index, together with mobility changes in Grocery & Pharmacy, Transit Station, and Workplaces displayed the best performances in predicting *R*_*t*_. In any case, climatic variables have been found to have a much weaker explanatory power compared to socio-economic and disease control factors (27).

In this work, we aim to assess the role of both mobility and seasonality on SARS-CoV-2 propagation in a rather infection-naive population by analyzing data for all peninsular Spanish provinces plus the Balearic Islands. We study this relation at a time period when the level of susceptibility to the disease was very high [above 80% during summer 2020, see Supplementary material and Instituto de Salud Carlos III (28)]. This is, before the massive vaccination campaign (see Supplementary material) and the appearance of the Omicron variant in Spain during the winter of December 2021 (29), which dramatically reduced the susceptible population. The outcomes of the 48 different provinces provide a nice natural experiment to check for the presence of correlations between COVID-19 growth rates and mobility/meteorological data. The reason is that, on the one hand, the criteria for the detection of cases and their protocols were uniform across the different provinces given the Spanish legislation, which mandated the different regions to report all COVID-19 cases as a *Enfermedades de Declaracion Obligatoria* (Compulsorily Notifiable Disease) in the national statistics (30). On the other hand, the different regions in Spain, known as Autonomous Communities in English or *Comunidades Autónomas* (CC.AA.) in Spanish, were the political entities in charge of deciding and implementing different non-pharmaceutical interventions. With the exception of a certain general ban on gathering, which was compulsory in all Spain during some short period of time, most measures affecting mobility were decided by the different regional governments (31, 32).

Another important reason to choose this analysis period is the COVID-19 infection rates. The data indicate that the different infection waves during this period (known as the second and third wave of COVID-19 in Spain) did not end due to a lack of susceptible individuals, but rather due to external factors. The evolution of the waves differed between provinces, but they always followed a similar pattern of a sharp increase in cases followed by a rapid decrease. This similar behavior in the two epidemic waves must be related to interactions between the population (mobility) and other factors (such as climate) to maintain different levels of growth at different times. Furthermore, data from the ENE-COVID-19 surveys (a national-level epidemiological study) not only shows that the level of people with prior immunity before September 2020 was very low (below 6.5% on average for the national level), but also during this period (September 2020–March 2021) this level increased only marginally, to around 13% on average for Spain (see Supplementary material) (28). These reasons, along with the previously mentioned initial vaccination coverage and the emergence of the new Alpha variant, seem to be sufficient arguments to reject any explanation associated with a lack of susceptible individuals to justify the change in the growth curve and to highlight the relevance of temperature and mobility in the epidemic dynamics.

Mobility data was obtained from Facebook Data for Good, which uses GPS mobile information, as well as from aggregated mobile phone antennae information, provided by the Spanish Ministry of Transport, Mobility and Urban Agenda or, in Spanish, *Ministerio de Tansportes, Movilidad y Agenda Urbana* (MITMA). Meteorological data was obtained by processing public satellite data. COVID-19 growth was processed from raw case counts from Instituto de Salud Carlos III, the institute in Spain that compiles and produces uniform and standard case counts of COVID-19. The high correlation between the different series of mobility and meteorological data requires a reduction of dimensionality, achieved using Principal Component Analysis (PCA). Univariate and multivariate correlations with different time-lags between mobility and meteorological data series and COVID-19 growth rate were assessed together with its robustness. We show that correlations are absent between the mobility/seasonal signal and the measure of epidemic growth at the same data, while very high correlations are present in all provinces at the expected time delays of 1 or 2 weeks. As a global assessment, COVID-19 growth showed a very high correlation with mobility data and a small but not negligible correlation with meteorological data, following the anticipated direction: lower mobility leads to slower growth, and lower temperatures result in faster growth.

## 2 Methods

**Figure 1:**
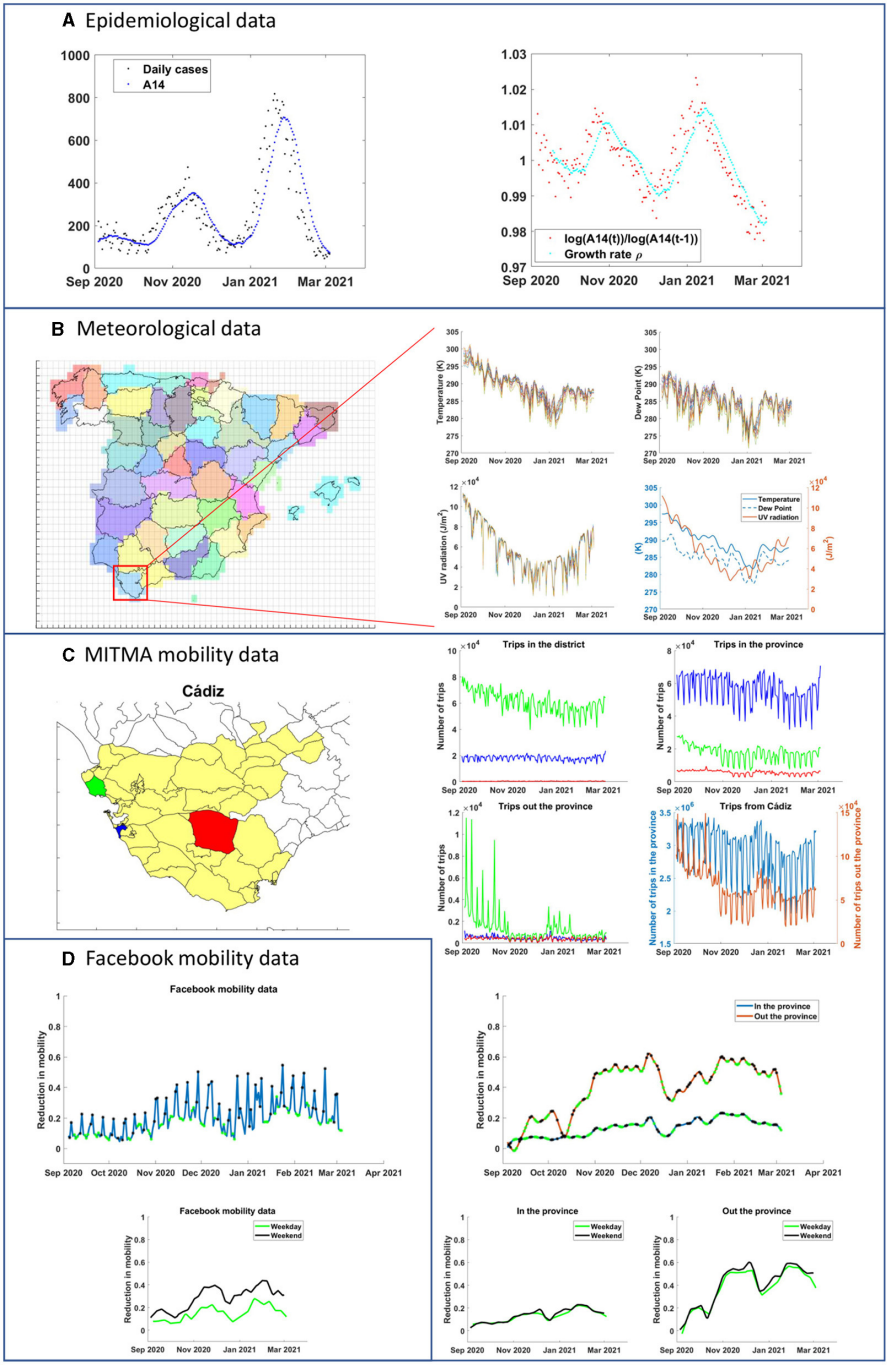
Schematics of processing in all the signals considered in the manuscript from 4/9/2020 to 4/3/2021 in all Spanish provinces. We use as an example the dataset from the province of Cadiz (South of Spain). **(A)** Left: case counts and biweekly average from Instituto de Salud Carlos III (public source). Right: computation of the local epidemic growth (red dots) as defined in Equation 2. **(B)** Left: scheme of the Spanish covering for satellite data and selection for Cadiz as described in Equations 3, 4. Right: Daily time series of temperature, UV radiation, and dew point, as well as the resulting smooth processing. **(C)** Left: district/area subdivision of Cadiz in MITMA data structure. As example, we use three subdivisions (green, red, and blue). Right: In each graph we show trips in the same subdivision, trips from the subdivision to any other in the province, and trips from each subdivision and a location out of province. The final graph shows the result of adding the previous data from all the subdivisions in the province. This amount of trips is then compared to the baseline mobility obtained from data obtained in February 2020 to compute, below, the reduction in mobility. Finally, weekday and weekend data series are split and interpolated with a spline function. **(D)** Original source of mobility in Facebook is already provided as a reduction. Only the weekend/weekday split is implemented, as with data from MITMA.

The political structure of Spain divides the country into 17 Autonomous Communities (*Comunidades Autonomas* or CC.AA.). Most of these CC.AA. are further divided into provinces, up to a total of 50 provinces. We investigate the evolution of COVID-19 epidemic and its relation to mobility and seasonal data in all provinces except for the two in the Comunidad Autonoma of the Canary Islands, given that mobility data from Facebook does not present complete data of the islands. The 48 remaining provinces encompass all the provinces of the Spanish part of the Iberian Peninsula plus the Balearic Islands (see Figure 1).

We consider the time series from 04/09/20 to 04/03/21 (181 days), encompassing the time span where most Spanish provinces were experiencing the second and third waves of the epidemic. We stop in March 2021 when vaccination could influence the evolution of the epidemic. After that, in the summer of 2021, tracking of key mobility data was discontinued, so we can not compare our analysis with and without vaccination. All data sets came from public sources but required important post-processing. A description of the process is shown in Figure 1.

**Figure 2:**
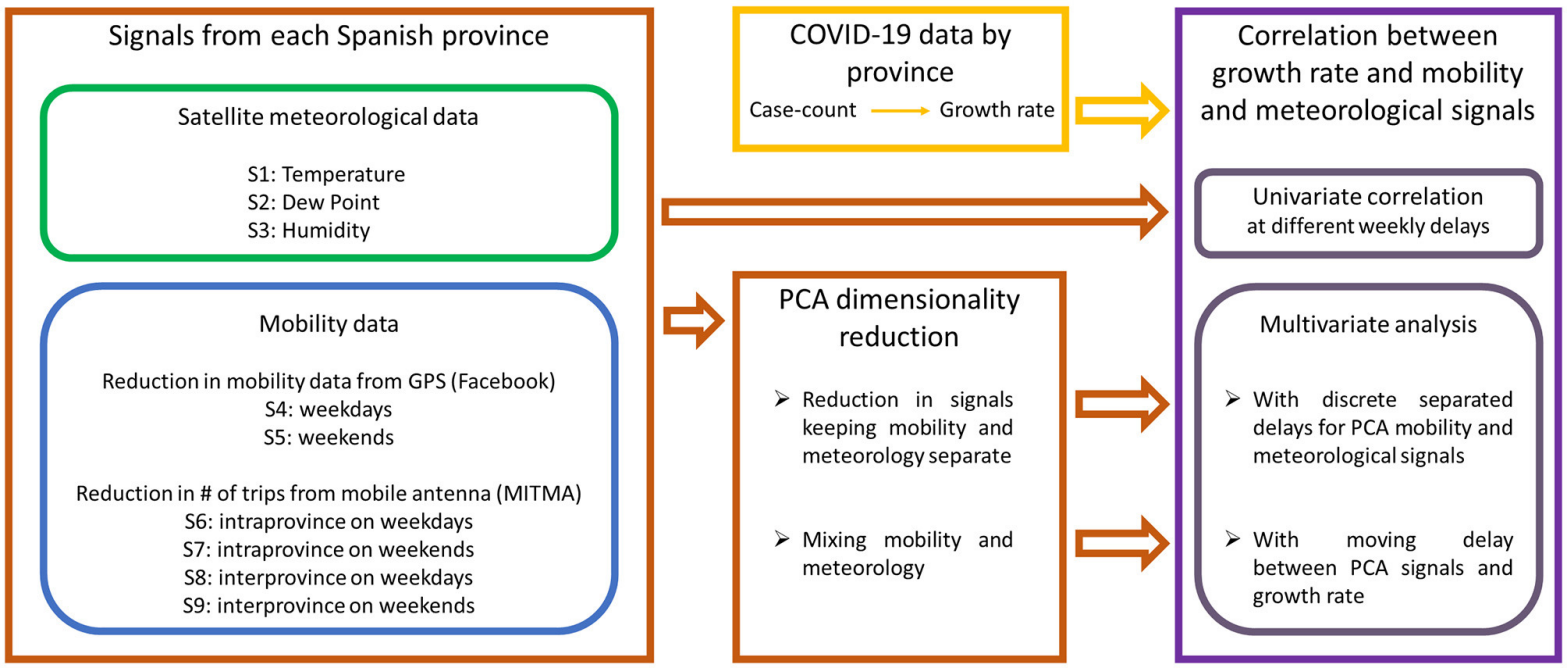
Schematics of the research framework pursued in this paper. We consider meteorological and mobility data series at province level **(leftmost box)** and COVID-19 case-count data from which we compute the growth rate **(upper middle box)**. We first compute univariate correlations between growth rate and each one of the signals independently at each province. This univariate correlation analysis is done at different time lags of 0, 1, and 2 weeks (as shown in the **upper right box**). Secondly, we perform two types of Principal Component Analysis (PCA) to the meteorological and mobility signals **(bottom central box)**. In one, we compute the principal components (PCs) of the meteorological signals separated from the PCs of the mobility signals. In the other, we compute the PCs with all meteorological and mobility signals together. Then, we perform a multivariate analysis of the growth rate with the resulting PCs **(bottom right box)**. For the case in which principal components are computed separately, we introduce a fixed delay of 2 weeks for meteorological data and 3 weeks for mobility data, as these are the delays that present highest univariate correlations. For the multivariate analysis of the growth rate with the PCs that mix all signals together, we perform a moving delay analysis to check which time delays present the highest correlation between the PCs of the signals and the growth rate. These multivariate analysis are done independently for each province, obtaining different highest correlation coefficients for different provinces.

We obtained six different mobility data sets from two sources: Facebook movement range data sets and individual travel information from the Spanish Ministerio de Transportes, Movilidad y Agenda Urbana (MITMA). We also obtained three meteorological data sets from satellite measures. All in all, nine data sets plus case-count information. We perform principal component analysis (PCA) of the six mobility data series and three seasonal data series in each province. We show that most of the information can be compressed into three mobility components and two meteorological components. If combined, PCA also shows that five series, mixing all signals, explain more than 95% of the variability. From this information, we construct two different sets of principal components, integrated and split, and use them to investigate the level of explanatory power on COVID-19 growth. A visual schematics of this research framework is provided in Figure 2.

### 2.1 Epidemiological data

Data of cumulative SARS-CoV-2 positives are taken from public repositories at Instituto de Salud Carlos III. From this, the daily number of new cases *x*(*i*) is obtained, where *i* indexes the day. To calculate the growth rate for each province, we first calculate the past 2-week average number of daily cases *A*14(*i*) for province *k* (Equation 1):


(1) $$\begin{array}{l} A14_{k}\left(i\right)=\frac{1}{14}\overset{i}{\underset{j=i-13}{\sum }}x_{k}\left(j\right). \end{array}$$

The log growth rate is then calculated and averaged across 2 weeks to smooth spurious daily patterns ρ, which is a well-known practice in European data case count [see for example Villanueva et al. (33)], as


(2) $$\begin{array}{l} \rho_{k}\left(i\right)=\frac{1}{14}\overset{i}{\underset{j=i-13}{\sum }}\log\left(A14_{k}\left(j\right)/A14_{k}\left(j-1\right)\right) \end{array}$$

The growth rate is computed with a backward average to eliminate any possible information about the future. This way, the growth rate at time *i* only includes information on case counts in the past. This is important to identify possible cause-and-effect explanations behind correlations. For example, a decrease in mobility/seasonal data on a given day must lead to variations in the COVID-19 growth signal necessarily in the future, given the time delays involved between infection and the detection of the case, among others. Then, with the above definition, any correlation between the COVID-19 growth cases and the mobility or meteorological data without a time delay is spurious. If there is any possible causal relation behind the correlation of both signals, a time-delayed analysis must be included. We explain this point in more detail in the following sections and in the discussion.

### 2.2 Meteorological data

Temperature, dew point, and UV data are obtained from the Climate Data Store of the European Union Copernicus Programme (34). They provide estimates of atmospheric quantities, such as the temperature and dew point temperature at two meters above the surface of the Earth or the downward UV radiation at the surface. These estimates are the result of data assimilation: the combination of previous forecasts and new observations made by the ECMWF (European Centre for Medium-Range Weather Forecasts). Data are given hourly and, spatially, every 0.25 degrees for both latitude and longitude. To relate this satellite data with seasonal information of each province, the space is divided into squares of 0.25 × 0.25 degrees, centered on the latitude and longitude points of the given data set, and it is determined, for each of these squares, which province is the dominant (which province is present the most in the corresponding square). Then, a temporal average across the day 24 h, and a spatial average across all Province squares, give the corresponding daily data for each one of the provinces.

Each of the surface squares *S*_*i*_ is associated with the province more present in the area. In some cases the square is fully within a province, but this is not the case near a border (see Figure 1B). So, the set of squares *S*_*i*_ that corresponds to a given province *p*, named *S*_*p*_, is mathematically defined as


(3) $$\begin{array}{l} S_{p}=\left\{S_{i}|S_{i}\cap A_{p}>S_{i}\cap A_{p^{\prime }},\forall p^{\prime }\in P_{\backslash p}\right\} \end{array}$$

where *P*_\*p*_ is the set containing all Spanish provinces except province *p* and *A*_*p*_ is the area of province *p*.

After defining the set of squares of the grid that encompasses a province, i.e., the squares from the set *S*_*p*_ of each province *p*, the meteorological signal of a province can be computed as the average of the signals of these squares. More formally, let the squares in *S*_*p*_ be numbered from 1 to *N*_*S*_*p*__. Then, the corresponding value of a given meteorological variable X for the province *p* and day *d*, $\bar{X}\left(p,d\right)$, is computed as


(4) $$\begin{array}{l} \bar{X}\left(p,d\right)=\frac{1}{24}\overset{24}{\underset{t=1}{\sum }}\left(\frac{\sum_{S_{i}\in S_{p}}X\left(S_{i},d,t\right)}{\left|S_{p}\right|}\right) \end{array}$$

where |*S*_*p*_| is the cardinality of set *S*_*p*_ and *X*(*s, d, t*) is the value of the meteorological variable *X* at the square *S*_*i*_ on day *d* and hour *t*.

### 2.3 Mobility data

Mobility data is obtained from two independent data sources, Facebook and MITMA. In these, mobility is measured in complementary ways, providing a unique opportunity to have accurate aggregated mobility data and interaction in a geographical area with a cross-comparison of methods.

Facebook movement range maps are provided by the program Facebook Data for Good. They aggregate GPS information from mobile devices that use the Facebook app and have the GPS tracking system active, as described in detail in (35). Each province is divided into level-16 Bing tiles (which are approximately 500 meters by 500 meters in Spain) and the amount of tiles visited on average per person in a given day is computed. Facebook does not provide the average number of trips, but rather its reduction (or increase) compared with equivalent days in February 2020. Figure 1 shows typical raw data for Cadiz as an example. For instance, a value of 0.2 indicates a reduction of the mobility of 20% compared with the equivalent day in February 2020. A negative value would indicate an increase in mobility. Therefore, a higher value of the signal indicates a larger reduction in the average number of tiles visited per person.

We observe a marked difference in the mobility data between weekdays and weekends. Mondays and Fridays also have slightly different behavior. In order to aggregate comparable data, we split the original data set into weekdays and weekends. Weekdays include all Tuesdays, Wednesdays, and Thursdays, except bank holidays, and the first day before and after a bank holiday. Weekends encompass Saturdays, Sundays, and bank holidays. In order to have a continuous data set, we interpolate and smooth the signal using the *csaps* function in MATLAB with a smoothing parameter of 0.05 so as to have a value for each day that captures properly the trend, at least in the same 2-week window frame that we use to compute the average growth of the epidemics. The smoothing procedure did not change significantly the outcomes, as long as the smoothing parameter was set around 0.2–0.02, averaging information below the 1-week scales. We also checked that increasing the smoothing parameter up to 0.5 did not change our results significantly.

The second source of mobility information is provided by MITMA and has its source in the geolocalization of more than 13 million mobile phone carriers. These devices record the nearest mobile tower each time the user employs his or her phone or each time that phone actively connects to an antenna. From these records, anonymized by the data source, MITMA captures the mobility patterns, dividing Spain into 2,850 areas (mostly municipalities or aggregations of them and districts for big cities) for that purpose, and computing the number of trips from one area to another and within the areas, every hour. A trip is defined as a detection which is more than 500 meters apart. We aggregate the public data both in time and space. We divide trips into internal and external to a particular province depending on whether the trip's initial and final positions are within MITMA areas that belong to the same province or not. We compute all trips within a province and those within/without of the province, constructing two data sets. From this, we calculate the reduction in mobility with respect to a baseline, taking the 7-day average mobility in the first week after February 21, 2020, as a reference. Notice that MITMA strictly measures the number of trips, while the data from Facebook can be better related to the total distance traveled in each trip. Each variable measures thus something slightly different providing a rather complete and detailed description of mobility.

As for Facebook data, we similarly observe the weekday/weekend separation, and we thus split the data as above. Again, all data series were subject to the same cubic-spline smoothing process. All in all, for each province there are 6 mobility measures. Facebook movement range on weekdays (FB WD), Facebook movement range on weekends (FB WE), MITMA trips within a province on weekdays (MITMA WD), MITMA trips within a province on weekends (MITMA WE), MITMA trips out/in of the province on weekdays (MIO WD), and MITMA trips out/in of the province on weekends (MIO WE).

### 2.4 Principal component analysis

**Figure 3:**
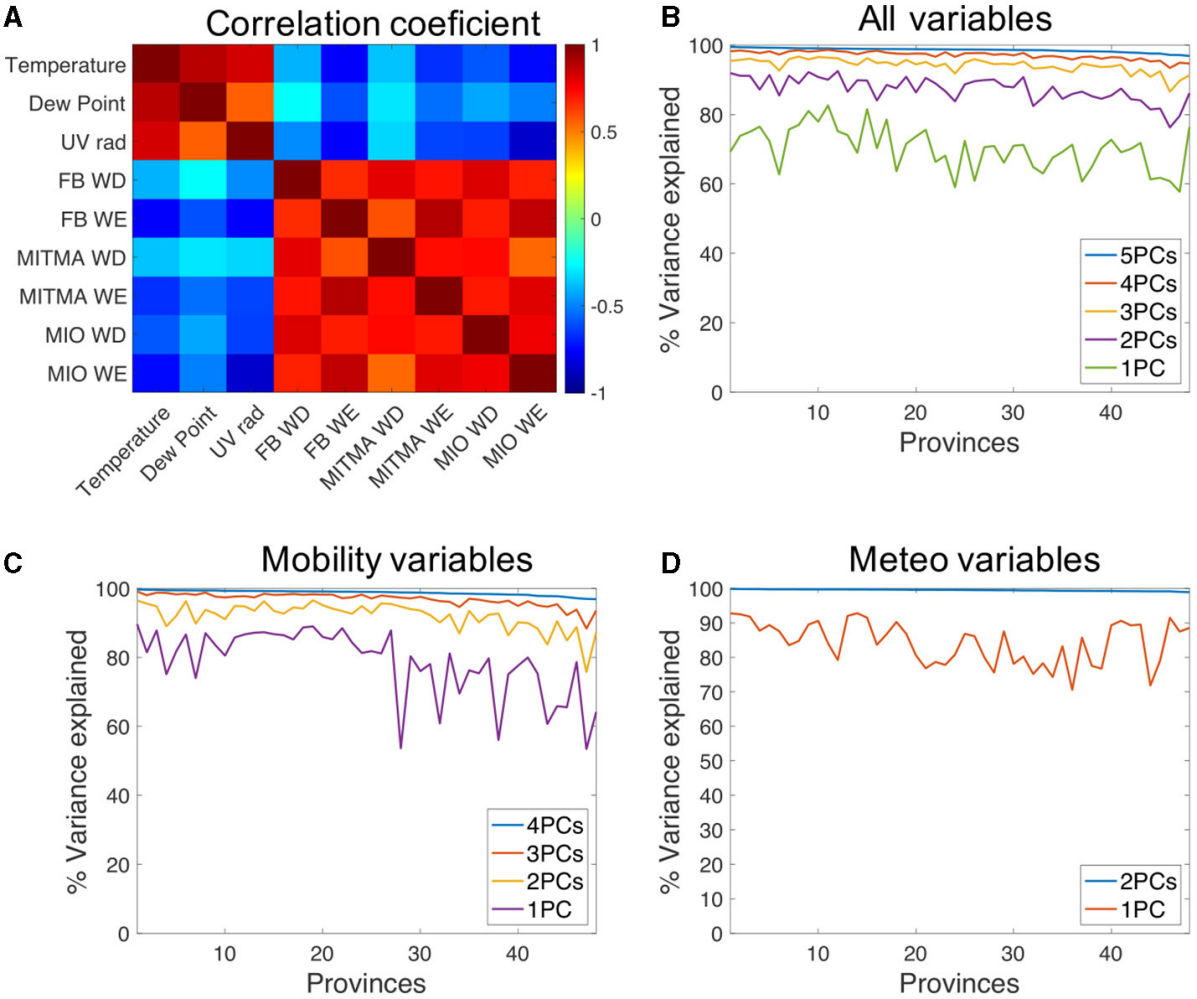
**(A)** Color-coded table with the average value of the correlation coefficient between the nine variables considered in this work for 48 provinces. **(B)** Cumulative percentage of the variance of the nine time series explained with different number of principal components in each province. **(C)** Same analysis as in **(B)** but only with six mobility data series. **(D)** Same analysis but with the three meteorological data series.

The different day-based time series defined previously are heavily correlated, as shown in Figure 3A. The pairwise average value of the correlation coefficient in all 48 provinces on average shows that the correlation is very high among different measures of mobility on weekdays and on weekends, but not so much crossing weekends and weekdays. This reinforces the idea of splitting the series of mobility between weekdays and weekends, as they convey different mobility information. This is critical during the period analyzed, since some restrictions in a number of provinces were activated only on weekends. Interestingly, there is also a correlation (or anticorrelation) between mobility and meteorological data (Figure 3). The higher the temperature the lower the decrease in mobility. Similarly, better weather conditions are correlated with a higher level of mobility, which is to be expected.

Given these correlations, we employ Principal Components Analysis (PCA) of normalized mobility/meteorological signals when performing multivariate analysis (see research framework in Figure 2). More specifically, we use PCAs on two different types of data. First, we consider a PCA of the full set of nine data series (integrated PCA). Secondly, we also use PCA for the six mobility time series on the one hand and for three meteorological on the other (split PCA).

### 2.5 Time-lagged correlation

Changes in mobility or in environmental variables do not affect immediately the evolution of the epidemics. To start with, there are structural epidemiological causes due to delays between infection and the registration of a case in the COVID-19 case count in each province. Then, there is a time-lapse until the development of symptoms, which can range from three days up to, in some cases, more than a week (36). There is also a time-lapse between the development of symptoms and the visit to a doctor, with the consequent registered diagnosis. So, at minimum, there must be a 5–7 day delay between mobility/seasonal signal and its effects on epidemic growth.

Furthermore, this delay between a possible cause and its effect could be larger. The growth of epidemics is not only determined by the mixing itself but also by the very level of incidence. All things equal, a higher level of incidence requires a higher level of mixing. Besides, there are second-round effects on any given infection process, where the first round of reduction of infections will produce further reduction down the road due to the lower level of infected people. To have a better idea of the expected time-lags in the correlation analysis, we use a SIR model where the number of susceptible and recovery rates are known from the literature, and the infection rate is deterministically fixed by an external signal. With this SIR model, we can then test how a causal relationship between a given signal and the infection rate translates into a correlation between the signal and the growth rate, as defined in this manuscript. We observe that, if there is no delay between infection and its reporting, the maximum correlations are typically obtained with time-lags around 1 or 2 weeks depending on the type of signal and parameters of the model (see Supplemental material for details). However, given the typical five/ten day delay between infection and report, we expect the largest correlations with a time lag between signal and growth rate of 2-3 weeks. This is why the analysis (Figure 2) must include these time delays.

It is important to stress that zero time-delays should not present any correlation with growth if there is a possible causality behind the correlation. On the other hand, correlations with a time-lag between 2 and 3 weeks can be expected. For this reason, in our univariate analysis, we analyze correlation coefficients with 0, 1, 2, and 3 weeks delays between the signals and the growth rate. Later, we use multivariate analysis between the PCs of the nine time series and epidemic growth. In this case, due to the mixture of signals, there might be different time-lags with respect to epidemic growth. In this situation, a more detailed spanning of the time-delays is used. We study different correlation coefficients as the time-lags are moved continuously between 7 and 28 days so that we can track the time-lag that produces the maximum correlation between these two values (see Figure 2).

### 2.6 Multivariate analysis

Given the notable correlation observed between the original signals (see Figure 3A in the Results section), we perform multiple linear regression in each province between the growth rate and the principal components of meteorological and mobility data to quantify the partial contribution of each variable into the reported epidemiological trends. More specifically, we infer the statistical associations between the COVID-19 case growth and the principal components (PCs) within each data type (meteorological or mobility data), and across both types (combined meteorological and mobility data), as described in Figure 2.

By using PCA within and across the time series of each data type, we performed in total four different multiple linear regression analysis. First, for each province *j*, we took the first two PCs ($X_{j}^{\text{PCMet1}}$ and $X_{j}^{\text{PCMet2}}$) of the three meteorological series as independent variables (Equation 5):


(5) $$\begin{array}{l} \rho_{j}\left(i\right)=\beta_{1j}X_{j}^{\text{PCMet1}}\left(i-\tau \right)+\beta_{2j}X_{j}^{\text{PCMet2}}\left(i-\tau \right)+\beta_{0j}, \end{array}$$

where ρ_*j*_(*i*) is the COVID-19 case growth rate of province *j*, at time *i* and τ is the time lag used for the analysis. We use τ = 0, 7, 14, and 21 days. For each one of the provinces, we obtain the coefficients β_1*j*_ and β_2*j*_ and the goodness of fit. Then, we repeated the same procedure for the first three components of the six mobility time series ($X_{j}^{\text{PCMob1}}$, $X_{j}^{\text{PCMob2}}$, and $X_{j}^{PCMob3}$) (Equation 6):


(6) $$\begin{array}{c} \rho_{j}\left(i\right)=\beta_{1j}X_{j}^{\text{PCMob1}}\left(i-\tau \right)+\beta_{2j}X_{j}^{\text{PCMob2}}\left(i-\tau \right)+\\ \beta_{3j}X_{j}^{\text{PCMob3}}\left(i-\tau \right)+\beta_{0j}. \end{array}$$

Finally, we consider linear regression models that simultaneously include mobility and meteorological PCs as regressors. In particular, we study two possible combinations of the regressors. The first model is to use the PCs obtained within mobility and meteorological data, each with its corresponding time lag (Equation 7):


(7) $$\begin{array}{l} \rho_{j}\left(i\right)=\beta_{1j}X_{j}^{\text{PCMob1}}\left(i-\tau_{\text{mob}}\right)+\beta_{2j}X_{j}^{\text{PCMob2}}\left(i-\tau_{\text{mob}}\right)+\\ \;\beta_{3j}X_{j}^{\text{PCMob3}}\left(i-\tau_{\text{mob}}\right)\\ \;+\beta_{4j}X_{j}^{\text{PCMet1}}\left(i-\tau_{\text{met}}\right)+\beta_{5j}X_{j}^{\text{PCMet2}}\left(i-\tau_{\text{met}}\right)+\beta_{0j}. \end{array}$$

Because of the observed amount of regressor co-linearity, it might be of interest in this model to disentangle the specific contribution of each data type conditioned on the other. To this end, we resort to the estimation of partial R-squared [$R_{p}^{2}\left(\cdot \right)$] (37) for mobility and meteorological data, respectively. The partial R-squared (also called the coefficient of partial determination) is the proportion of variance explained by a given subset of regressors over the dependent's variable variability that is not explained by the remaining regressors. Hence, it accounts for the unique and added contribution of the given subset of regressors with respect to the remaining set. More formally, let SSE^full^ ≡ SSE(*X*^1^, *X*^2^, ⋯ , *X*^*M*^) denote the residual sum of squares of the above model Equation 8 when considering the entire set of possible *M* regressors and let $\text{SSE}\left(X^{i_{1}},X^{i_{2}},\cdots \;,X^{i_{n}}\right)$ be the SSE for a regressors' subset of size *n*, where *n* < *M*. Then, for a given province *j*, the partial R-squared of the mobility PCs is estimated as (37):


(8) $$\begin{array}{l} R_{p}^{2}\left(X_{j}^{\text{PCMob1}},X_{j}^{\text{PCMob2}},X_{j}^{\text{PCMob3}}\right)=\frac{\text{SSE}\left(X_{j}^{\text{PCMet1}},X_{j}^{\text{PCMet2}}\right)-{\text{SSE}}^{\text{full}}}{\text{SSE}\left(X_{j}^{\text{PCMet1}},X_{j}^{\text{PCMet2}}\right)}. \end{array}$$

Similarly, the partial R-squared of the meteorological PCs may be estimated as (Equation 9):


(9) $$\begin{array}{l} R_{p}^{2}\left(X_{j}^{\text{PCMet1}},X_{j}^{\text{PCMet2}}\right)=\frac{\text{SSE}\left(X_{j}^{\text{PCMob1}},X_{j}^{\text{PCMob2}},X_{j}^{\text{PCMob3}}\right)-{\text{SSE}}^{\text{full}}}{\text{SSE}\left(X_{j}^{\text{PCMob1}},X_{j}^{\text{PCMob2}},X_{j}^{\text{PCMob3}}\right)}. \end{array}$$

To overcome regressors' co-linearity, an alternative model is to use the leading PCs of the nine original time series altogether (Equation 10), that is, $X_{j}^{\text{PCm}}\left(i\right)$, where *m* = 1, 2..., *M* indexes the first, second,..., *M*-th principal component as regressor:


(10) $$\begin{array}{l} \rho_{j}\left(i\right)=\overset{m=M}{\underset{m=1}{\sum }}\beta_{mj}X_{j}^{\text{PCm}}\left(i-\tau_{j}\right)+\beta_{0j}. \end{array}$$

In this latter analysis, we consider up to a maximum of five regressors *M* = 5 since they had been shown to explain most of the total variance in the original signals. Furthermore, we assume that for a given province, the time lag τ_*j*_ is fixed across all PCs. We note that the fitted time lags might slightly vary across provinces but they are expected to be narrowly distributed if causation between mobility/meteorological variables and growth rate underlies the measured correlations. Overall, this model allows for a simple decomposition of the total R-squared into the sum of each separate regressor's R-squared. Instead, the interpretation of the analysis outcomes in terms of PCs with mixed meteorological and mobility information might be challenging.

## 3 Results

### 3.1 Principal components

As explained in the previous section, we compute PCs either separately from the six mobility and the three meteorological times series (split PCA), or obtain mixed PCs from all nine time series (integrated PCA). In both cases, most information is contained in just five time series. Figure 3B illustrates, for each province, the fraction of the variance explained by an increasing number of components for the integrated PCA. The first five components explain more than 95% of the variance. In fact, four components explain more than 90% in all provinces. Figure 3C shows the same analysis but for the mobility time series where reducing from 6 to 3 time series, in all provinces, conserves more than 90% of the total variance. For a PCA with only meteorological information, the three time series can be reduced to 2, explaining close to 98% of the total variance as shown in Figure 3D. All in all, the integrated PCA seems to reduce more efficiently the information contained in the original time series. From the variance explained we conclude that two components are relevant for meteorological data, and three for mobility. When integrating all signals, four or five PCs explain most of the variance and, thus, are the ones significant.

**Figure 4:**
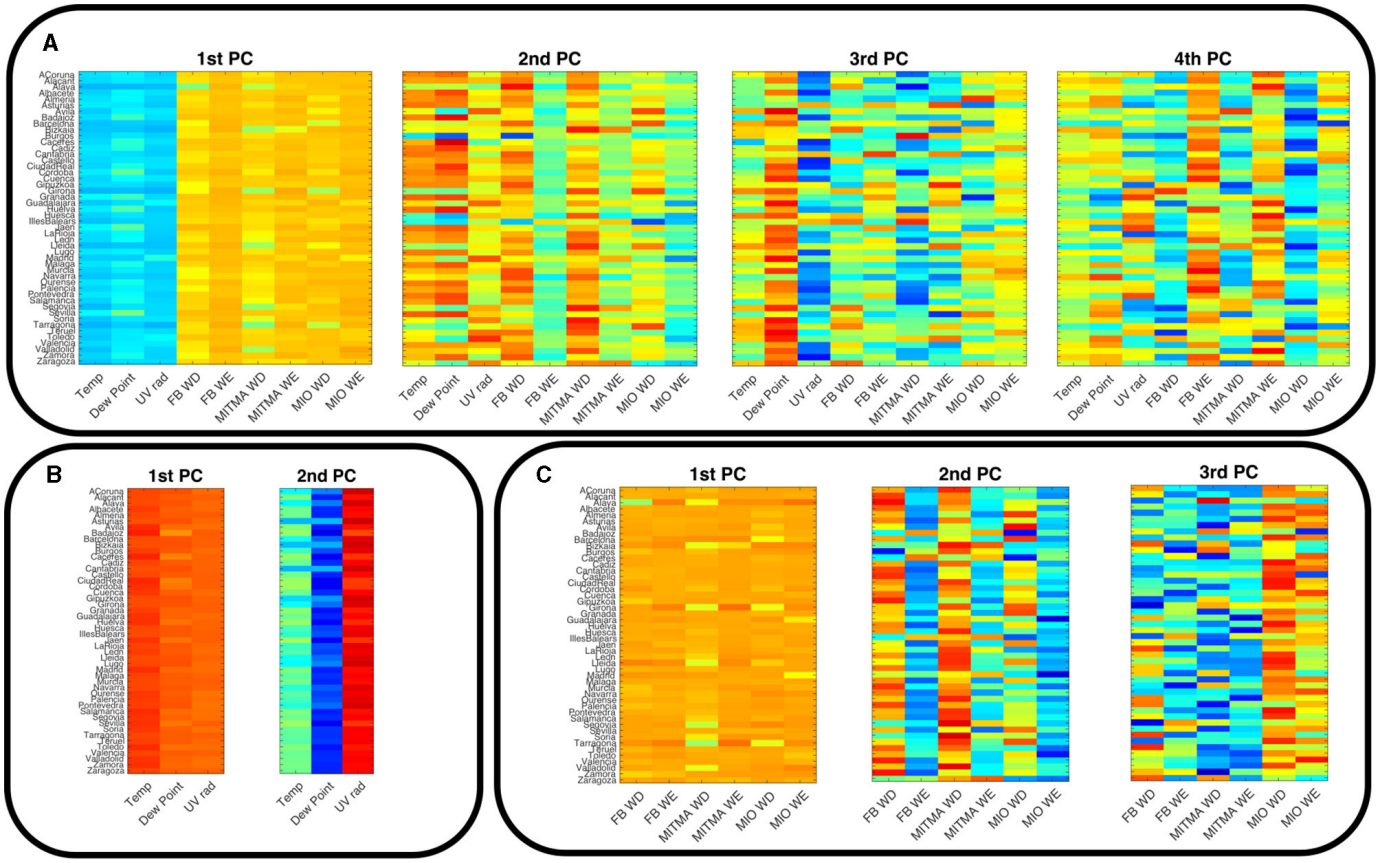
Color-coded coefficients from the PCA in each province for **(A)** the first, second, third, and fourth PCs of the nine data time series considered together; **(B)** first, second, and third PCs of the six mobility data series **(C)** first and second PCs of the three meteorological data series.

Even if the integrated PCA reduces more efficiently dimensionality than the split PCA, we keep also the latter to help with the interpretability of the results. This can be understood by analyzing how the different principal components weigh the different signals in each province. For the integrated PCA where all nine signals are introduced, we find that weights of the first PC in each province are nearly equally distributed among the different variables, with different signs due to anticorrelation between variations in mobility and in Temperature/DewPoint/UV. The coefficient corresponding to each signal is color-coded in the left graph in Figure 4A for each province. The second component, also in the panel, generally weighs more heavily meteorological data series together with weekday mobility time series, neglecting the weekend mobility time series. There are, however, important exceptions in Girona, Toledo, Tarragona, or Avila. In the third component, there are more exceptions, but generally, a strong separation of Dew Point from UV radiation (large coefficients with different signs) joins an important weight of the in/out weekend time series. The last graph in Figure 4A shows that the fourth component presents a clear mix of mobility and meteorological signals that is very different from province to province. There is a general split between labor and weekend mobility, but with very different weights on Dew Point and UV radiation depending on the particular province. The fifth component, not shown, is similarly dependent on province without a clear pattern although there is a tendency to split Facebook labor mobility from MITMA with different relative weights for meteorological variables. Table 1 shows the average value of each of the nine coefficients of the first four principal components for the 48 Spanish provinces.

**Table 1:** Coefficients of the first four PCs with respect to all the variables, averaged over the 48 provinces.

	**Temp**	**DP**	**UV rad**	**FB WD**	**FB WE**	**MITMA WD**	**MITMA WE**	**MIO WD**	**MIO WE**
1st PC	–0.3313	–0.2621	-0.3300	0.3070	0.3721	0.2745	0.3568	0.3342	0.3704
2nd PC	0.3335	0.3248	0.2664	0.3663	–0.0404	0.4093	0.0926	0.1958	–0.0713
3rd PC	0.1418	0.4576	–0.2744	0.0684	0.0423	-0.1803	–0.0122	0.0556	0.2114
4th PC	0.1376	0.1502	0.0642	–0.1816	0.3821	-0.0839	0.2953	-0.2992	0.1166

The PCA split for mobility and meteorological time series leads to a more consistent picture of each component, see Figures 4B, C. The two first PCs of the meteorological time series are simple to interpret, the first one is an average of all the time series, while the second component splits UVB radiation on the one hand and Dew Point on the other, with a smaller contribution of temperature (see Supplementary material). Mobility interpretation is more complex. As shown in Figure 4C, the first component represents an average of all mobility signals across all provinces, the second component splits the weekday, and weekend data and the third component splits trips In/Out of the province measured by MITMA from the rest. We notice that some provinces have these patterns for the second and third components interchanged. Tables 2, 3 display the average values of the coefficients for the main components of meteorological and mobility data respectively, showing this clear interpretation.

**Table 2:** Coefficients of the first two PCs with respect to the meteorological variables, averaged over the 48 provinces.

	**Temp**	**DP**	**UV rad**
1st PC	0.6252	0.5570	0.5452
2nd PC	–0.0928	–0.6355	0.7475

**Table 3:** Coefficients of the first three PCs with respect to the mobility variables, averaged over the 48 provinces.

	**FB WD**	**FB WE**	**MITMA WD**	**MITMA WE**	**MIO WD**	**MIO WE**
1st PC	0.4006	0.4187	0.3628	0.4245	0.4032	0.4155
2nd PC	0.3387	–0.3166	0.4541	–0.1281	0.1087	–0.3319
3rd PC	0.0090	–0.1740	–0.2329	–0.2668	0.4381	0.2242

To sum up, joining all time series to perform a single integrated PCA reduces the effective dimension very efficiently to four, maximum five relevant signals. The interpretation of the fourth and fifth components, however, is not straightforward. On the other hand, when the separation between signals is kept, the resulting time series from the PCA have a clearer interpretation, uniform across-provinces. We present in the following sections how the signals resulting from both types of PCA reductions in dimensionality correlate with epidemic growth and how robust these correlations are to changes in PCA analysis.

### 3.2 Univariate analysis

**Figure 5:**
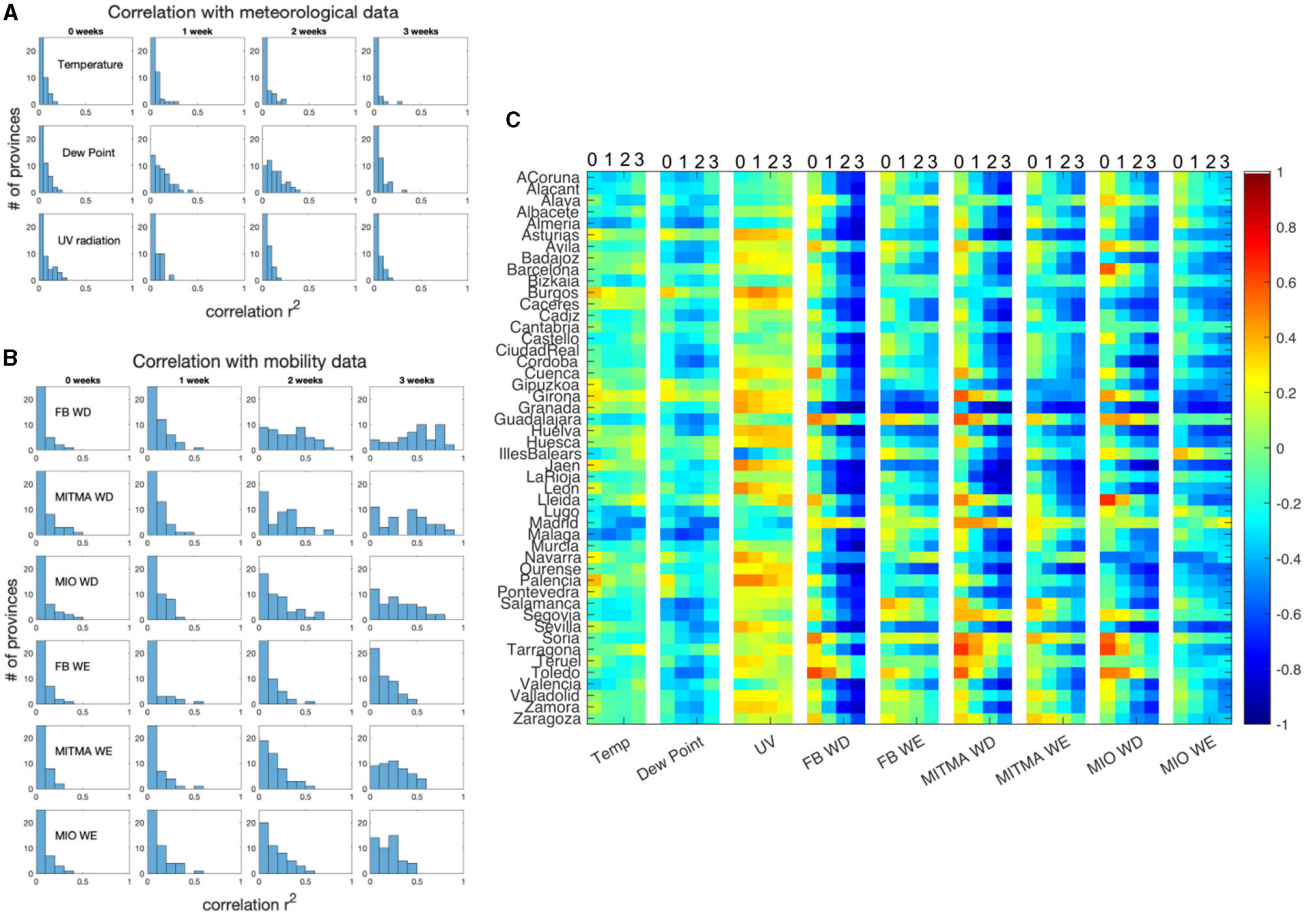
**(A)** Histograms of R-Squared values from the univariate correlation analysis between each meteorological time signal and the growth rate in COVID-19 cases for 48 Spanish provinces. Four different histograms are shown for each time signal corresponding to four different time delays between the meteorological signal and COVID-19 growth rate. **(B)** Equivalent histograms for each of the univariate analyses using mobility data. **(C)** Color-coded Pearson coefficient of the univariate correlation between the growth rate of COVID-19 cases for each province and each one of the nine time signals. For each time signal, four values of the Pearson coefficient are shown corresponding to 0, 1, 2, and 3 week delays between signals.

First, we compute the correlation between the nine time series (three meteorological and six related to mobility) and the infection growth rate for all Spanish provinces with four different time delays (from 0 to 3 weeks), as described in the methods section. That is, for each time series and for each time delay we obtain 48 different coefficients of determination. Figure 5 presents histograms of these coefficients for each univariate analysis. In Figure 5A, for example, the first row shows the correlation between the average temperature time series and the growth rate, with the four different time lags. It is clear that this correlation is very low in all provinces. A similar pattern is observed with UV radiation in the third row. This can also be seen in Table 4, where the median over all provinces is shown. The only exception is the appearance of a certain correlation between the Dew Point and the growth rate with 1–2 weeks delays. Still, the R-squared does not reach levels above 0.5. It also demonstrates that correlation at zero delay is negligible, as discussed in the methods section.

**Table 4:** Median values over all provinces of R-squared of the correlation between different signals and the growth rate.

	**Temp**	**DP**	**UV rad**	**FB WD**	**FB WE**	**MITMA WD**	**MITMA WE**	**MIO WD**	**MIO WE**
*R* ^2^	0.0179	0.1145	0.0383	0.5275	0.1240	0.4455	0.2272	0.2721	0.1948

The meteorological and mobility variables are taken with a time lag of 2 and 3 weeks with respect to growth.

Figure 5B shows the same histograms for univariate analysis between growth rate and mobility time series. Here, correlations are generally larger. Their maximum occurs with time lags between 2 and 3 weeks, roughly 1 week later than for meteorological variables. The highest correlations are obtained between growth rate and labor day mobility time series. Table 4 presents the median coefficient of determination using a 2-week delay lag for meteorological data and 3-week lag for mobility data. Mobility data have across the board higher level of correlation than meteorological data. The median values reach 0.6 for the correlation between Facebook weekday mobility and the growth rate and 0.5 for the mobility data output from MITMA.

Figure 5C displays color coded Pearson coefficients of the correlation between the growth rate in COVID-19 cases and each of the nine signals in each province. For each signal, four different time delays are shown. As discussed above, correlations with meteorological time series are very low, except for the dew point temperature in some provinces, but when present, the correlation is negative, meaning the lower the temperature the higher the growth rate. Correlation, as indicated, is higher between COVID-19 growth rate and mobility when using 2–3 week time delay. Pearson coefficients are negative indicating that the lower the reduction in mobility (higher mobility) the higher the growth rate in COVID-19 cases.

This last figure also shows that a high correlation, however, is not present systematically over all provinces. Madrid and Guadalajara, two central neighboring provinces, for example, present a very low correlation in all univariate analysis between COVID-19 growth rates and mobility. Similarly, some of the provinces with the lowest population densities, like Teruel and Soria, present very low correlation coefficients.

### 3.3 Multivariate regression analysis between COVID-19 growth rates and the principal components of mobility and meteorological signals

**Figure 6:**
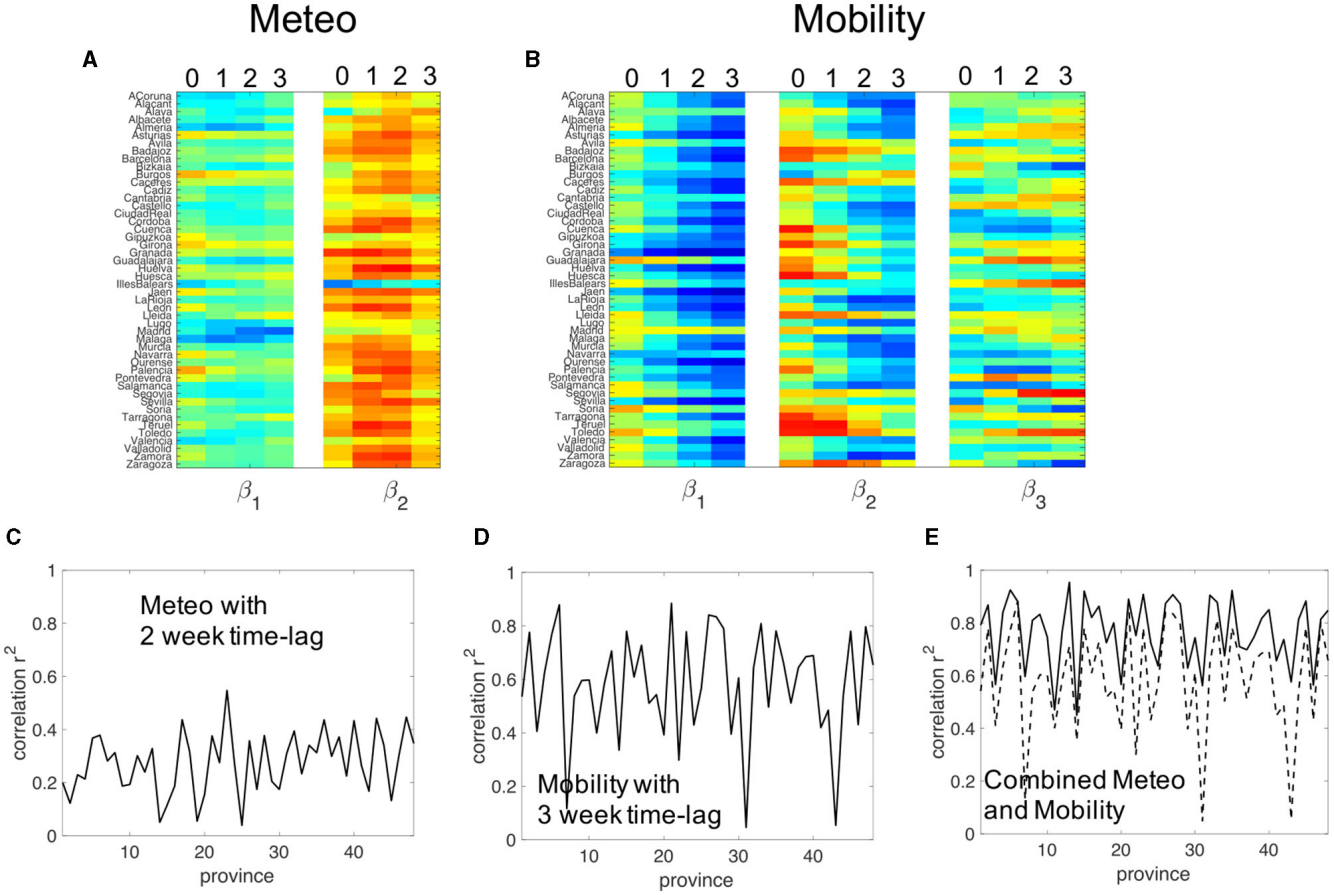
**(A)** Color-coded coefficients β_1_ and β_2_ for the multivariate regression between the COVID-19 case count growth rate and the two principal components (PCs) of the meteorological time series using four different time lags between the time series: 0, 1, 2, and 3 weeks. **(B)** Color-coded coefficients β_1_, β_2_, and β_3_ for the multivariate regression between the COVID-19 case count growth rate and the three PCs of the mobility time series using four different time lags between the time series: 0, 1, 2, and 3 weeks. **(C)** Goodness of fit for each province of the multivariate analysis with the two PCs of the meteorological data time series with a 2-week time lag. **(D)** Goodness of fit for each province of the multivariate analysis with the three PCs of the meteorological data time series with a 3-week time lag. **(E)** Goodness of fit for the multivariate analysis between the COVID-19 growth rate and five time series, the two PCs of the meteorological time series plus the three PCs of the mobility series using 2 and 3-week lags respectively.

We start the multivariate analysis by studying the correlation of the COVID-19 case-count growth rate ρ with the three principal components of the mobility data series, on one hand, and with the two principal components of the meteorological data series on the other. We obtain first the multivariate linear coefficients for different time lags between the components and the growth rate, as indicated in the methodology. Figure 6A shows the first and second coefficients β_1_ and β_2_ color-coded for each province. Four values of β_1_ and β_2_ are shown, corresponding to no time-lag and time-lags of 1, 2, and 3 weeks between the growth rate and the two principal components of the meteorological data series. In Figure 6B, the same but for the three coefficients β_1_, β_2_, and β_3_ in the same analysis for the three principal components of the mobility data series.

The graph shows that, for zero time lag, coefficients are roughly zero across all provinces. The goodness of fit, not shown, is close to zero as expected. Coefficients deviate from zero when the delay is about 1 or 2 weeks. This is the earliest effect possible between a potential cause and effect as we will discuss later in more detail. Coefficients are negative for the first component and positive for the second. For mobility, large coefficients appear with a 3-week delay, with the corresponding β_1_ being highly negative, as expected if a larger reduction of mobility leads to a lower growth rate of the disease propagation.

We then focus on time lags of 2 weeks between meteorological data and growth rate, and 3 weeks between mobility data and growth rates. Figure 6C shows the R-squared for all provinces using 2-week time delays between the growth in case counts of COVID-19 and meteorological time series. We observe that, with few exceptions, *R*^2^ is systematically lower than 0.4, and in some provinces, it falls below 0.2. Figure 6D shows that the goodness of fit is much higher for the correlation between COVID-19 growth and mobility. Most provinces have *R*^2^ larger than 0.6. Interestingly, a few provinces do present a very low correlation. For example, Teruel, one of the less densely populated provinces in Spain, along with Madrid and Guadalajara (a neighboring province of Madrid that has strong mobility interactions with the capital) present *R*^2^ values below 0.3.

In principle, both mobility/interactions and environmental factors affect growth rate, so the next step is to check the correlation when both data series are combined. Figure 6E shows the R-squared coefficient of a multivariate analysis between the growth rate of COVID-19 cases and the 2 meteorological plus 3 mobility PCs, using a 2-week time lag between growth rate and meteorological time-series and a 3-week delay between growth rate and mobility data. Combining both, a remarkable result arises. The goodness of fit raises significantly in all provinces. There is no longer any province with a low goodness of fit. Most provinces exhibit a goodness of fit above 0.8 and, almost all above 0.6.

We have checked that this increase is not merely due to the increase in the number of time series in the multivariate analysis. Figure 6E shows that adding two random time series to the principal components of the mobility time series increases the goodness of fit marginally. This implies that, while meteorological data on its own, does not present important correlations, when it is added to mobility data it provides relevant complementary information.

### 3.4 Relative contribution of mobility and meteorological variables to the epidemics growth

The average and median values of R-squared for the previous multivariate analyses are shown in Table 5. It is of special interest to focus on the R-squared value between the growth of COVID-19 cases and the five time series (two meteorological plus three mobility) that provide this high correlation over all Spanish provinces. The median *R*^2^ value is 0.81. It is interesting to compare this value with the square value of the five weights β_*i*_ shown in the Supplementary material. While the principal components of mobility and meteorology are by construction not correlated among them, the principal components of mobility can present correlations with those from meteorology. In this sense, the quadratic sum of the weights is not equal to *R*^2^.

**Table 5:** Average and median value of the coefficient of determination (R-squared) for several of the multivariate analyses performed.

	**2 PC meteo**	**3 PC mobility**	**3 PC mobility + 2 PC meteo**
Average *r*^2^	0.28	0.58	0.77
Median *r*^2^ [Q1–Q3]	0.29 [0.19–0.36]	0.60 [0.47–0.77]	0.81 [0.69–0.87]

The time lags used are 2 weeks with the principal components of the meteorological time series, and 3 weeks with the PCs of the meteorological time series.

**Figure 7:**
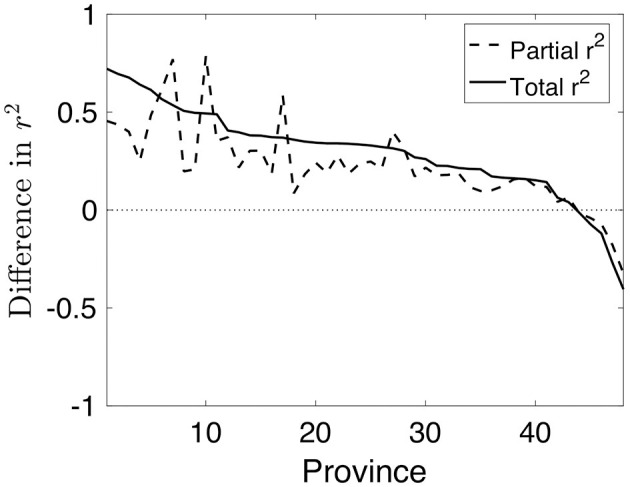
In solid line, difference between the R^2^ of the mobility and meteorological variables. The dashed line correspond to the difference of the partial *R*^2^.

In this context, we wish to provide a fair comparison between the individual explanatory power of meteorological and mobility information. To this end, we consider three PCs for meteorological and mobility time series, respectively, to avoid any bias in the analysis due to differences in the number of regressors. Then, we follow two approaches. First, we analyze two separate models, one for meteorological data and another for mobility time series and compute the corresponding R-squared in each province. Finally, we represent the difference in R-squared between mobility and meteorological data for each province in decreasing amounts (see solid line in Figure 7). Due to the reported correlation between the PCs of each data type, the R-squared of each separate model captures a fraction of shared explanatory power by meteorological and mobility variables. Hence, in order to account for the amount of COVID-19 growth variability that each data type explains uniquely, we compute the partial R-squared of each subset of PCs in a joint model including three meteorological and three mobility PCs, respectively. The partial R-squared of the meteorological (resp. mobility) signals computes the added contribution of these variables to explain the variance unexplained of the dependent variable (growth) when only mobility (resp. meteorological) signals are present in the model. Finally, we represent the partial R-squared difference for each province in Figure 7 (see dashed line) superimposed to the above-mentioned R-squared difference. Interestingly, both curves consistently show positive differences (>0.2, medium effect size threshold) in a large ensemble of provinces, while negative differences (<−0.2) in a much smaller subset. This suggests that in the majority of provinces the relative contribution of mobility into COVID-19 growth is stronger than the contribution of meteorology because it also explains a great deal of epidemiological variability that is not associated to meteorological variables.

### 3.5 Robustness analysis. Multivariate analysis with principal components mixing mobility and meteorological data

We check now the robustness of this high correlation between the growth rate of COVID-19 case counts and a proper combination of meteorological and mobility data. To this aim, we consider all nine signals and compute the different principal components for each series. Most of the information in the variance is collected in the first four signals, more than 90% in all provinces, with marginal improvement in the next two components (see Figure 3B and Section 2.4). We proceed to compute now a multivariate linear regression of the growth rate of COVID-19 case counts with the first four principal components of the time series.

As described in the Method Sections, different provinces present slightly different mixtures of the signal in each component because there are some correlations or anticorrelations between mobility and meteorological data. This implies that the delay between the signals and their effects are necessarily different in each province. To test that correlations are indeed high, we look for different time lags between the principal components and the growth case count of COVID-19.

**Figure 8:**
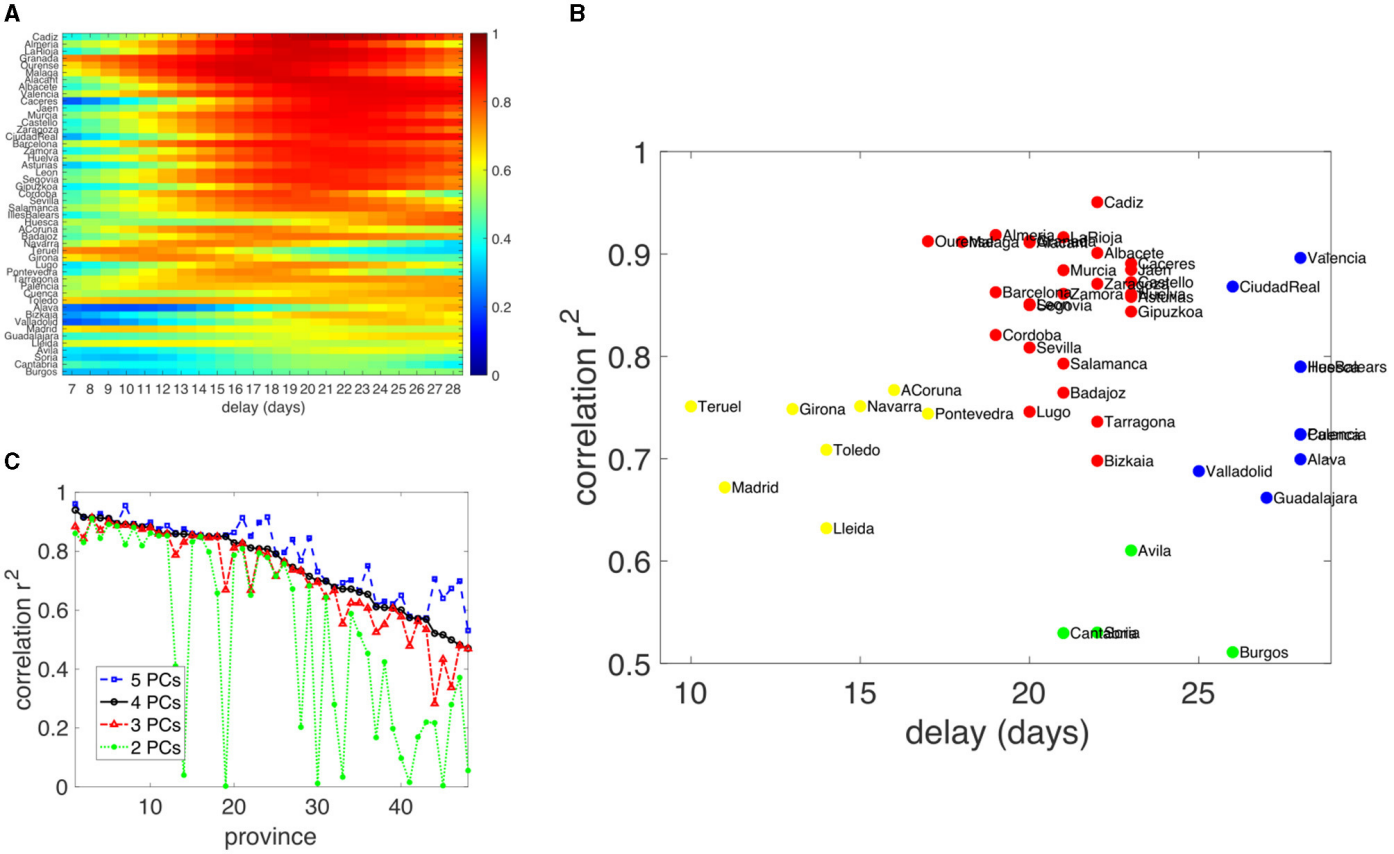
**(A)** Color-coded value of *R*^2^, for each province, of the multivariate linear regression of the four PCs of the mobility and meteorological time-series in front of the growth rate in the case count of COVID-19. The X-axis presents the different time lags used between the PCs and the epidemic growth rate. **(B)** Shows a scatter plot where the maximum goodness of fit is plotted against the time delay used for this maximum. **(C)** Shows the highest *R*^2^ for each province when two, three, four, or five PCs are used in the multivariate analysis. Provinces are sorted from higher to lower correlation using four PCs.

Figure 8A shows in color code, for each province, the R-squared as a function of the time-lag indicated in the X-axis. Most of the provinces present very high *R*^2^ for time delays between 15 and 25 days. As we will show in the discussion, 10–14 days is the minimum time lag that must be present between cause and effect. Figure 8B presents a scatter plot of the time lag with the best goodness of fit for each province. A simple cluster algorithm (38) indicates the presence of four groups of provinces. Those with a very high correlation cluster around 3-week delays (red dots). A second cluster has an intermediate high *r*^2^ at 0.65–0.85, but presents the maximum correlations sooner, around 2 weeks (yellow), a third one with rather larger correlations but with a maximum correlation at longer delays (blue) and a final cluster of slightly lower correlation (*r*^2^ at 0.6–0.8) which seems to present time delays at slightly more than 3 weeks (green).

A very important result is shown in Figure 8C. The *R*^2^ for the time lag is shown in Figure 8B using a different number of PCs in the multivariate analysis. The graph shows how the correlation levels in each province do not change much once the three first PCs are used in the multivariate analysis. More than ten provinces sharply increase the correlation when three PCs are used. Adding more components still produced an improvement, but more marginally. Seven provinces do present a significant increase in the correlation with five components compared with four, but the increase is not as broad-based as when three PCs are used instead of four. It is highly possible that when third components are included, further information simply overfits without any more meaningful explanatory power. In order to further test the robustness of this analysis, we perform the same multivariate analysis using a common 3-week delay between all PCs and the growth rate. Table 6 shows that the median R^2^ does not change appreciably. In any case, when information from four time series is included the lowest quartile is always above, and the median is not affected.

**Table 6:** Average and median value of the coefficient of determination (R-squared) in the multivariate analysis using four PCs.

	**4 PC all series (common delay)**	**4 PC all series (adapted delay)**
Average *r*^2^	0.75	0.79
Median *r*^2^ [Q1–Q3]	0.79 [0.66–0.88]	0.80 [0.69–0.87]

In one case for all time series we use a common time delay of 3 weeks, while in the other we use a province-by-province time delay that presents the higher coefficient of determination.

## 4 Discussion

In this article, we have demonstrated that a very high level of correlations between the growth rate and the principal components of mobility and meteorological data is sustained under different analyses and scenarios in all Spanish provinces. More than half of the provinces present a high R-squared above 0.85, with all provinces above 0.5. In fact, with the exception of one province, R-squared is always above 0.6 when using 4 PCs with a province-adjusted optimum time delay. We consider this to be a reliable indicator of the actual correlation between meteorology, mixing, and growth rate of the epidemics since all signals are obtained through well-established protocols.

First and foremost, the growth rate of cases is based on the detection by health services at both primary care and hospital/ICU admittance levels. Cases detected in the hospital carry considerably more delays than those detected in primary care. However, in the national register, cases are provided according to the date of diagnosis. Another important point is that, during the period analyzed, the level of detection of cases in Spain was very high, most of the symptomatic cases and an important number of asymptomatic cases were detected (see Supplementary material). An important fact is that the evolution of cases in each province is very different. Some provinces have a first large wave in November followed by a small one in January. Others have similar waves in November and in January, and others have mainly one wave in January. There is a wide spectrum of outcomes, which makes it possible to hypothesize on the causal origin of correlations, as we will discuss later.

Regarding meteorological data, most provinces have a rather similar structure of the evolution of temperature during this period: It first decays rather linearly from the beginning of September until late December, then a major drop in temperature happens due to the storm Gloria that affected Spain during January 19–25 and finally a return to the low-temperature levels typical for February. For mobility data, the fact that we have information from two complementary sources with very different inputs (Antenna and GPS sources) provides a coherent and trustworthy description of how people responded to legal non-pharmacological interventions and the changes in behavior affected by the news provided by the media. In this sense, it is a very good surrogate of very different effects that can affect the mixing of people and drive the epidemics. The fact that different provinces had different non-pharmacological interventions at different times (39) and with different effects is key in our interpretability analysis. So, we have an important asymmetrical shock, very useful to test if this signal can have an effect on the circulation of the virus. On the other hand, the time series of mobility during weekdays and weekends are rather different from province to province (see the graphs in the Supplementary material). It is no surprise that, with such big differences in growth but a similar temperature profile, most of the correlations between purely meteorological time series and growth are very low. Temperature, at least at this stage of a very high susceptible population does not seem to play a major role in transmission on its own.

Our results point in the direction that mobility is either directly causal or highly directly correlated with other measures that directly affect the propagation of the disease via mixing when the population is highly susceptible. Other papers that use mobility data to make short-term predictions of the effective reproduction number (Rt) (40) have shown similar results. From data in Poland, Turkey, and South Korea (41) it has been shown that while the stringency index was associated with mobility data of the same day, mobility changes were associated with the number of cases 1 month later. Another study found that daily new COVID-19 cases in Spain are directly related to mobility habits 14 days before (42). Meteorological patterns are less relevant than mixing effects in the propagation of an epidemic with a large number of susceptible populations. This is consistent with the observation that warm and wet climates seem to reduce the spread of COVID-19, but these variables alone could not explain most of the variability in disease transmission (43).

In our data, we observe a very high correlation at the expected time-lags (42), especially when some information on the temperature is added to the principal components of mobility. However, the explanatory power of these time signals deserves a careful analysis regarding their interpretability, which we proceed to address. It is crucial to understand that correlation does not directly imply causation. We must delve deeper into whether a direct model with explanatory power using causal inference such as the one discussed above can be useful in future stages of the pandemic.

**Figure 9:**
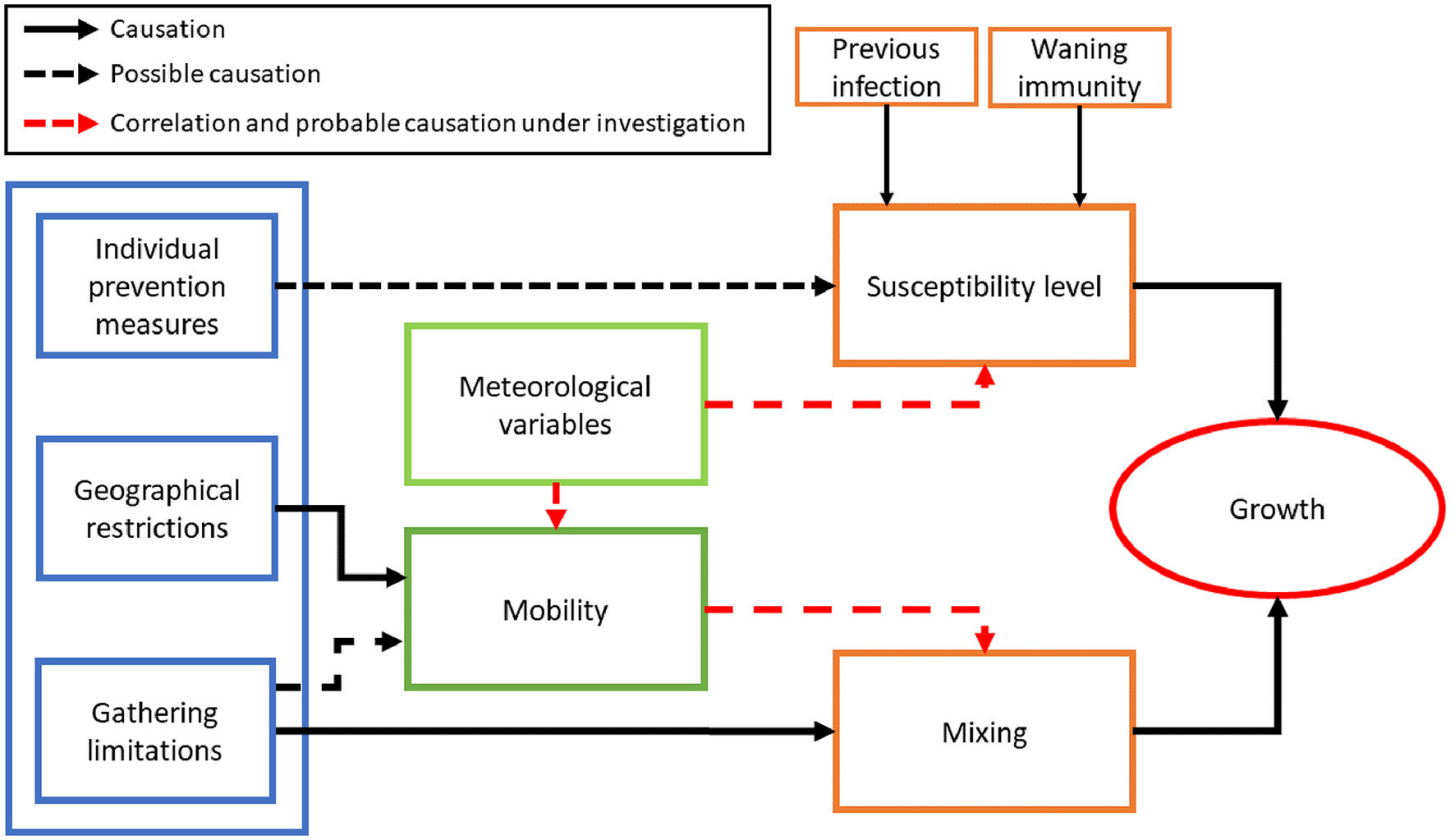
Schematics of the different relation between non-pharmacological measures (left in blue boxes), the observable measured in the data time series (green boxes) and its causation, possible causation, and correlation with the different features (in orange boxes) that are known to affect the growth of the epidemic. More details in the discussion.

Figure 9 helps to guide this discussion. On the right, the growth of cases is our measured output. Epidemic growth is well known to depend causally on, first, the amount of susceptible population. The smaller the number of susceptible people, the less ability the virus has to circulate. This, in our case, depends mainly on the level of previous infections, the specific variant under circulation, and the waning of its immunity, given that vaccination was not available at that point. Secondly, growth is directly linked in a causal form to the level of mixing. The fewer people interact with each other, especially in environments where high viral loads are possible, the more difficult it becomes for the disease to propagate.

Besides this clear causal link between susceptibility and mixing with epidemic growth, another two well-known causal relations have been established: a subset of non-pharmacological interventions clearly affects mixing, such as the prohibition of large crowds or prevention of gatherings with more than a given number of people. The question here, rather than on causality, is about efficiency regarding the relevance of the measure, given its costs. But for our purposes, it is clear that a population-level lockdown prevents the propagation of epidemics. Similarly, non-pharmacological measures that impose lockdowns or prevent travel between different geographical areas directly affect mobility, which is drastically reduced (44). Yet, other non-pharmacological interventions might also affect mobility even when no direct prohibition of movement is established. Heavy restrictions on gatherings on weekends or during certain times of the day at specific places can affect the level of mobility by limiting the available leisure activities of the population. For example, an NPI that prevents gatherings of more than 10 people in the same bar during the weekend might affect weekend mobility (45). Given that this effect is indirect and is not fully understood, we mark it as possible causality.

Two other possible causal relations have been discussed often in the literature. First, the possible effect of temperature and humidity in changing the susceptibility level of the population, whether directly, as it affects the ability of the nasal mucous membrane to prevent infection (46), or indirectly, because it reduces the environmental ability of the virus to remain in the air (47). While this causal link seems quite well-established and relevant for the flu, its relevance can not be extrapolated for a disease such as COVID-19 where a large part of the population was infection-naive. The second possible causal relation involves non-pharmacological interventions that aim to individually protect people, such as masking, which might reduce the effective susceptibility of the population reducing the viral loads that a person receives. So far, the causation is very clear in laboratory experiments, but it is clearly not as strong at the population level (48).

With this scheme in mind, our analysis focuses on the discontinuous lines indicated in red in Figure 9. Testing the explanatory power and correlation between environmental variables and growth tries to check if a causal relation is possible behind the correlations between temperature and growth. Our results point out that this causal inference might exist. Still, it is clearly the most dominant one when other non-pharmacological interventions are present and when the level of susceptible people is high. We cannot interpret that mobility directly causes the presence of large correlations, but points to the possible fact that the effects of non-pharmacological interventions, although indirect, are much stronger than those that aim to reduce the level of effectively susceptible people. Some studies have also suggested that the direct (negative) correlations between temperature and growth rate could be suppressed by the effect of mobility, since a higher temperature typically increases mobility, that in turn has a positive effect of the growth rate (49).

It is important to stress that this picture was valid before vaccination brought broad-based immunization. Later, it is possible that, as the number of naive people diminished to very small values, the effects of non-pharmacological interventions diminished in their relevance, as personal immunity increased. Still, our analysis provides highly valuable insight in the case of new epidemics. In this sense, we do not aim to address the question of the relevance of mixing/mobility limitations on the virus transmission during the latter stages of the pandemic, which has been debatable.

Finally, we have not included in the diagram the changes in susceptibility levels that occur when a major change in the virus variant appears, resulting in higher transmissibility. In the period analyzed, the same major variant was present (B.1.177 variant), except for the appearance of the Alpha variant in different parts of Spain in February and March. Supplemental material shows the evolution of the rate of alpha variants in the surveillance program carried out in the different Autonomous Communities. In most provinces it has a minor effect at the very end of our period, in others, it might have an effect during February. To test that the appearance of the Alpha variant does not have relevant effects in our arguments on correlation and causation, we have repeated our analysis eliminating the date from February from the correlation analysis, and the picture that emerges remains the same. Some provinces might change a bit the time lag with the highest correlation and, on average, the *R*^2^ between the growth rate and the PCs drops slightly for all, as expected.

## 5 Conclusion

In this article, we use Spanish data from 48 Spanish provinces to study how COVID-19 growth correlates with meteorological and mobility variables. We do not observe systematic large correlations with any meteorological data, but we do observe important correlations with the principal components obtained from the mobility time series, although not in all provinces. When combined, there is a sharp increase in the correlation levels. We observe this pattern of large and important correlations when we use a mixture of mobility and meteorological data with just three-four data series. Remarkably, only three or four time series produce such large correlations in the multivariate analysis of all 48 provinces. Overall, we find that mobility has a larger contribution to the growth rate than meteorological variables in most provinces, emphasizing the clear relevance of mobility for the propagation of the disease.

## Data availability statement

Publicly available datasets were analyzed in this study. This data can be found here. Mobility data can be obtained from MITMA (www.mitma.gob.es) and from Facebook's Data for Good (dataforgood.facebook.com). The meteorology data can be accessed upon request to the Copernicus Climate Change Service (cds.climate.copernicus.eu).

## Ethics statement

Ethical approval was not required for the study involving humans in accordance with the local legislation and institutional requirements. Written informed consent to participate in this study was not required from the participants or the participants' legal guardians/next of kin in accordance with the national legislation and the institutional requirements.

## Author contributions

DC: Conceptualization, Data curation, Formal analysis, Investigation, Methodology, Software, Validation, Writing – review & editing. VL: Conceptualization, Data curation, Formal analysis, Investigation, Validation, Writing – review & editing. TG: Conceptualization, Data curation, Writing – review & editing. AT: Conceptualization, Formal analysis, Methodology, Writing – review & editing. CP: Conceptualization, Formal analysis, Funding acquisition, Methodology, Supervision, Writing – review & editing. EA-L: Conceptualization, Data curation, Formal analysis, Investigation, Methodology, Software, Supervision, Validation, Writing – original draft, Writing – review & editing. BE: Conceptualization, Data curation, Formal analysis, Investigation, Methodology, Software, Supervision, Validation, Writing – original draft, Writing – review & editing.
