# Supplementary material for: A mixture of mobility and meteorological data provides a high correlation with COVID-19 growth in an infection-naive population: a study for Spanish provinces
Source: Front Public Health. 2024 Mar 7;12:1288531. doi: 10.3389/fpubh.2024.1288531 (PMC10962055; doi:10.3389/fpubh.2024.1288531)
Supplement: Supplementary file 1 [file Data_Sheet_1.PDF]

Supporting Information for ”A mixture of mobility and meteorological data provides a high correlation with COVID-19 growth in an infection-naive population: a study for Spanish provinces”

David Conesa[1], **Víctor López de Rioja**[1], **Tania Gullón**[2], **Adrià Tauste Campo**[1], **Clara Prats**[1], **Enrique Alvarez-Lacalle**[1], **and Blas Echebarria**[1,*]

[1]Physics Department, Universitat Politècnica de Catalunya - BarcelonaTech (UPC), Barcelona, Spain.

[2]Spanish Ministry of Transport, Mobility and Urban Agenda (MITMA), Madrid, Spain.

[*]blas.echebarria@upc.edu

Introduction

This supplemental material includes:

- S1 Overview of the initial Spanish vaccination coverage in 2021 with Figure SF1.
- S2 Seroprevalence of SARS-CoV-2 surveys (ENE-COVID) from June to November 2020 with Tables ST1 and ST2.
- S3 Estimation of COVID-19 detection and naive population in the Spanish population in March 2021 with Figures SF2 and SF3, and Table ST3.
- S4 The emergence of the Alpha variant in Spain in early 2021 with Figure SF4.
- S5 SIR model to estimate correlation time-lags between external series and growth rate with Figure SF5.
- S6 Figure of the incidence of COVID-19 and its growth rate in Spanish provinces from 4th of September 2020 to 4th of March 2021 (Figure SF6).
- S7 Figure of the evolution of the reduction in mobility from Facebook and MITMA data sources. (Figure SF7).

- S8 Figure of the temperature, dew point and UV radiation from the Copernicus Climate Data Store. (Figure SF8).

S1 An overview of the initial Spanish vaccination coverage in 2021

In Spain, as in many other European countries, the national COVID-19 vaccination campaign began at the end of 2020. On December 27, 2020, the Spanish government initiated the first phase of the COVID-19 vaccination campaign, administering the initial doses to high-risk and vulnerable individuals including residents of care homes, caretakers, and essential workers such as doctors.

This study compares mobility and meteorological data with the growth (incidence or rate) of COVID-19 prior to the availability of vaccines. To do so, a homogeneous scenario over an appropriate time period is required. On one hand, nationwide confinement in Spain ended in June 2020, with some exceptions in certain areas and times. On the other hand, vaccines became available towards the end of 2020, but not for everyone immediately. Therefore, vaccines were gradually administered to a portion of the population, but steadily (similar to other European countries). It's important to note that the effect of vaccines is not immediate, with early studies indicating 95% efficacy after 7 days from the second dose, with a 21-day interval between the first and second dose[1].

We define a level of vaccination that avoids any biases when determining the chosen time interval in the study. Figure SF1 shows the ratio of individuals vaccinated (with one or two doses) to the total population of all the regions in Spain in the study, known as Autonomous Communities in English or *Comunidades Autónomas* (CC.AA.) in Spanish, based on official data from the municipal register *Padrón*, obtained from the Spanish National Institute of Statistics (INE). The data is also presented in the form of a bar graph for the entire country.

In the first week of February 2021, only 2.7% of the Spanish population was vaccinated (1.2% had received the full regimen of two doses). This was not likely to have affected the results of the study. By the first week of March, 6.2% of the total population had received one or two doses of the vaccine (2.8% had completed the full course of vaccinations), with only one province (Asturias) exceeding 8% (less than 5% of whom had completed the regimen).

By the first week of April, over 11% of the population had been vaccinated, with 6% having

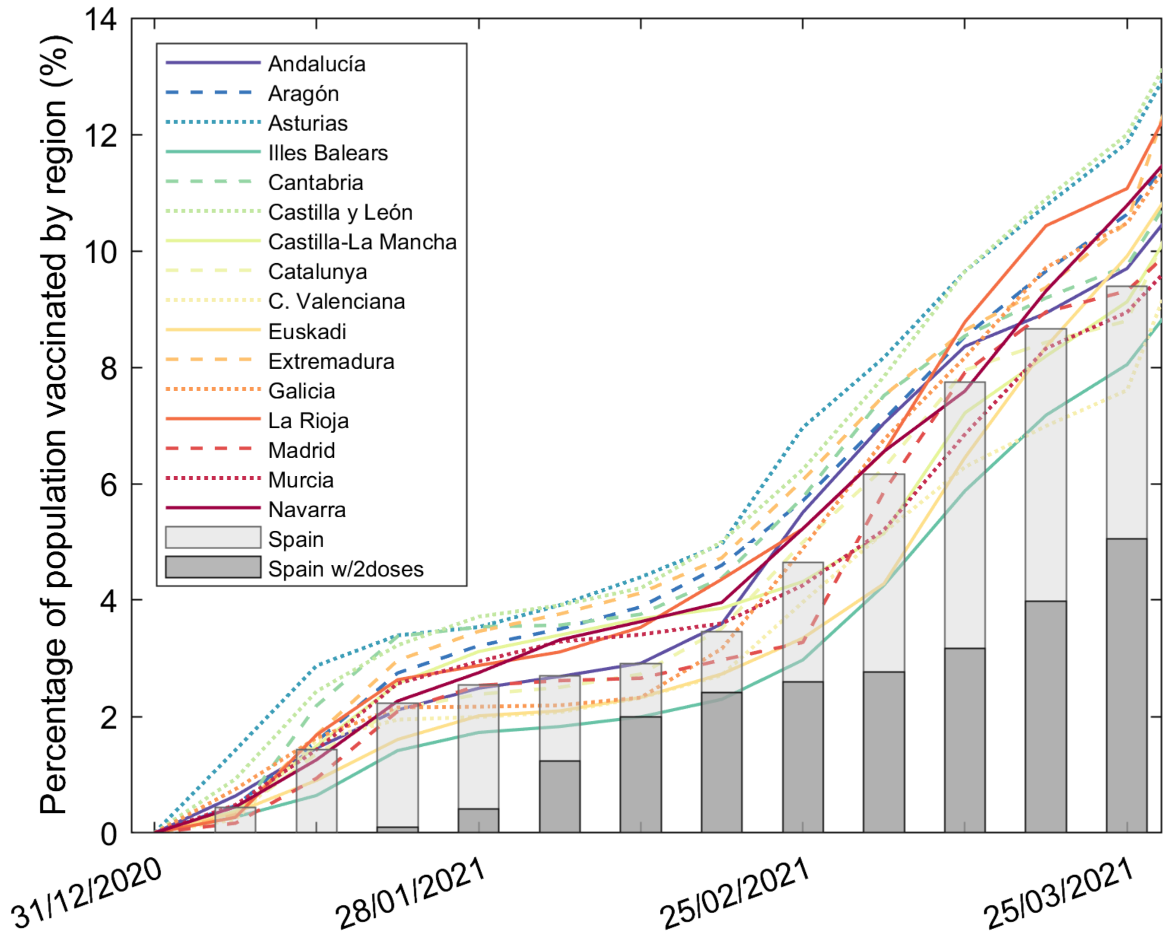

Figure SF1. Percentage of the population vaccinated with one or two doses of Pfizer, Moderna or AstraZeneca by the autonomous community (CC.AA.). The bar graph illustrates the overall percentage of the Spanish population vaccinated with either one or two doses (represented by clear grey) and those who received the full regimen (two doses, represented by dark grey). The same y-axis applies to all data.

received both doses. While this data may suggest the effect of the vaccines on the correlation between growth and mobility or meteorological data, it is important to note that the highest efficacy is achieved around a week after receiving the second dose. Therefore, the study's cutoff for the start of the vaccination strategy in Spain is set for the first week of March 2021, when less than 3% of the total population (and only specific population groups) had received the full recommended regimen. The study period covers the post-confinement period, from August 2020 to the start of the vaccination strategy, when its impact on population immunity was still negligible.

S2 Seroprevalence of SARS-CoV-2 surveys (ENE-COVID) from June to November 2020

Studying the seropositivity of the population (ratio of people who have specific antibodies against a certain infectious or pathogenic agent, in this case, against the SARS-CoV-2 virus) allows for a highly reliable assessment of the evolution of the pandemic in a territory. ENE-COVID (”Estudio nacional de sero-epidemiología de la infección por SARS-CoV-2 en España”, or National study of sero-epidemiology of SARS-CoV-2 infection in Spain) is a large Spanish sero-epidemiological study, based on the population, whose objectives are exactly this, to estimate the prevalence of SARS-CoV-2 infection and assess its temporal and spatial evolution[2,3]. Most data can be found in the study, but, ”In order to make this paper as self-contained as possible,” we have preferred to also show it here, along with explanations to justify our decisions.

The first phase of ENE-COVID, with its 3 rounds (27/04 - 11/05, 18/05 - 01/06 and 08/06 - 22/06 of 2020), included 68,287 participants. This first phase encompasses the end of the first wave of COVID-19 in Spain and the slow start of the second. The second phase or round 4, and the last of the study, (16/11 - 29/11 of 2020), involved up to 51,409 people from the total of the first phase. The first three rounds are very similar, as they were carried out coinciding with the start of the de-escalation (end of curfew restrictions) and in very close periods of time, in which, in addition, there was little transmission of the virus. Round 4, more than 3 months after the last analysis, shows the expansion of the virus in the midst of the second wave, caused mainly by the B.1.177 variant. These data give us, therefore, a rigorous study of a part of the population distributed throughout the Spanish territory with monitoring for a long period of time, between two waves (rounds 1, 2, and 3) and right in the middle of a wave (round 4).

In order to give the study complete meaning, we have emphasized that the level of the susceptible population must not be the main driving force for ending an epidemic wave. It is thus necessary to see that at the beginning of the analysis (August 2020) there is no group immunity in Spain and that the level of susceptible people was very high. Put another way, the ratio of people infected (seropositive test) during the first rounds (end of April - end of June 2020) and who have not died in August 2020, is a relatively low percentage.

First wave (Spring 2020)

To know who was infected during the first wave, it would be necessary to study the seropositive of rounds 1, 2, or 3. We do not have this directly from the reports. Therefore, we have devised two approaches to solve this: (i) from the global seroprevalence of the 4 rounds minus the seroconversion of negatives from the first phase (rounds 1, 2 or 3) to positives in the second phase (round 4); and (ii) from the seroconversions of rounds 1 to 2 and 2 to 3, which will be added to the seroprevalence of round 1. The results are very similar, so we have taken the average with a larger error interval. It should be noted that as we want the global or accumulated seroprevalence of the first three rounds, it is not necessary to take into account seronegativity, i.e. those people who had a positive test in one round and in later rounds the test is negative, as we would be decreasing the final result.

Table ST1 shows the global prevalence, i.e. the accumulated prevalence in these 4 rounds in ENE-COVID by province (ordered by CC.AA.). This estimator represents the percentage of the Spanish non-institutionalized population that, according to the data obtained with the rapid test, has had contact with SARS-CoV-2 since the beginning of the pandemic. In this case, we are only interested in the first phase (rounds 1-3), but we deemed it appropriate to include the data from the second phase (round 4) to see how, effectively, they are higher than the previous ones, as one would expect due to the second wave. With regard to the first three rounds, for Spain, the global prevalence is less than 6.5%, (almost half of the prevalence estimates for rounds 1-4). This estimator shows clear geographical variability: much of the provinces in the center of the peninsula have prevalences similar or higher than 15% (such as Cuenca, Soria, Madrid...). Above or around 9% is the entire central nucleus, with Castilla-León and Castilla-La Mancha, as well as some distant provinces such as Araba/Álava and Barcelona. Only provinces on the coastline (and therefore further from the central part) such as Coruña (extreme northwest of the peninsula), Asturias (extreme north), Tarragona and Murcia (northeast and southeast) and Huelva to the extreme southwest, as well as the Balearic Islands, have accumulated prevalences below 3%.

We want to emphasize our point here. Only a small fraction of the population, less than 6.5%

of the total, showed antibodies against SARS-CoV-2 (i.e., those who had been infected) and could potentially spread the virus, at least in the initial months of the study. Therefore, we can assert that the data used is not biased due to the immunity acquired by a small part of the population during the first wave.

Second wave (Autumn 2020)

The fact that the ENE-COVID study has lasted over time, allows us to estimate the incidence of seroconversions, in this case, new infections between the first phase of the study (rounds 1-3) and the second phase (round 4).

The results for Spain as a whole show that 3.8% (95% CI: 3.5-4.1) of the population has gone from being negative in the first phase to positive in the second phase. This means the number of people who were infected at some point between the end of June 2020 and the end of November 2020. This period is important because the first wave has disappeared and, on the other hand, we are in the midst of the second wave. The 3.8% is important as it reflects the proportion of the population infected exclusively during this second wave of the pandemic. Table ST2 also shows the seroconversion rates for each province. The geographical distribution differs from before, with percentages much higher in the center of the peninsula, but now it is much more spontaneous. Lleida, in Catalunya (*Catalonia*), stands out as the only province with a seroconversion rate >8%, but many others follow between 6% and 8%, all in the northern half of the country. The central provinces of Madrid and Guadalajara and Segovia (both attached to the capital) also stand out, which already had high prevalences in the first phase and whose increase is close to or higher than 5%. Finally, Galicia (extreme northwest of the peninsula) stands out, just the opposite, where its four provinces are those with the smallest seroconversion rate of all, between 0.9% and 1.7%.

With these low percentages of new infections during the second wave (round 4 is at the end of November, it must be taken into account that the second wave will last until the end of December 2020), it is difficult to think that the vast majority of the population will become infected and immunized, thus causing the number of cases to drop sharply. Therefore, other factors must have contributed to the decrease in the second wave's peak, indicating that the number of infections (and population immunity) during the second wave will not skew our results.

		Global or accumulated seroprevalence								
		Round 1-3					Round 1-4			
CC.AA.	Province	% (i)	% (ii)	CI (95%)			Number	%	CI (95%)		
Andalucía	Almería	4,3	3,8	2,0	-	7,7	722	6,7	4,5	-	9,7
	Cádiz	3,2	3,0	1,7	-	5,7	1004	6,1	4,6	-	8,1
	Córdoba	2,8	3,6	1,9	-	7,2	893	4,9	3,6	-	6,6
	Granada	4,5	3,9	2,2	-	7,0	854	9,6	7,2	-	12,8
	Huelva	2,0	2,3	0,9	-	5,8	831	4,7	2,7	-	8,1
	Jaén	4,6	5,4	3,3	-	8,6	805	8,2	5,8	-	11,5
	Málaga	3,8	4,9	2,9	-	8,7	1033	6,4	4,4	-	9,4
	Sevilla	3,1	3,3	2,0	-	5,5	1510	8,4	6,1	-	11,4
Aragón	Huesca	4,6	5,2	2,5	-	10,7	635	8,6	6,2	-	11,8
	Teruel	2,7	6,4	3,2	-	12,1	570	9,7	7,1	-	13
	Zaragoza	6,1	6,2	4,3	-	9,2	1131	12,7	10,1	-	15,8
Asturias	Asturias	2,1	2,3	1,4	-	4,1	1616	6,1	4,7	-	7,7
Balears, Illes	Balears	4,0	2,5	1,7	-	3,7	891	6,3	4,8	-	8,2
Cantabria	Cantabria	3,9	5,1	3,0	-	8,5	1371	6,3	4,8	-	8,4
Castilla y León	Ávila	9,6	9,4	6,2	-	14,8	592	14,9	11,4	-	19,2
	Burgos	7,3	6,5	3,9	-	11,0	706	12,4	8,8	-	17,1
	León	7,7	7,5	4,6	-	12,0	851	9,7	6,4	-	14,3
	Palencia	8,6	11,2	6,4	-	19,4	669	13,6	10,3	-	17,7
	Salamanca	9,3	10,0	6,5	-	15,4	752	13,5	10,5	-	17,2
	Segovia	10,4	13,1	9,3	-	19,4	584	15	11,2	-	19,7
	Soria	15,7	17,2	12,7	-	24,3	652	18,6	14,7	-	23,2
	Valladolid	8,4	8,9	5,4	-	14,5	947	11,4	8,4	-	15,3
	Zamora	7,7	8,4	4,7	-	15,5	606	13,9	10,4	-	18,4
Castilla-La Mancha	Albacete	12,8	13,0	9,3	-	18,5	904	17,1	13,3	-	21,8
	Ciudad Real	11,0	12,4	9,0	-	17,4	949	14,8	11,4	-	19,1
	Cuenca	15,2	15,2	11,5	-	20,6	660	18,7	15	-	23,1
	Guadalajara	12,1	12,2	8,4	-	18,0	621	17	13	-	21,9
	Toledo	11,7	10,1	5,9	-	17,5	833	15,4	11,8	-	19,7
Catalunya	Barcelona	8,3	8,8	7,0	-	11,2	2807	12,4	10,2	-	15
	Girona	4,4	4,5	2,8	-	7,5	787	11,4	8,4	-	15,4
	Lleida	4,0	4,2	2,5	-	7,7	564	12,2	7,7	-	18,7
	Tarragona	1,8	2,0	1,0	-	4,5	659	5,6	3,6	-	8,7
C. Valenciana	Alacant	3,9	3,9	2,6	-	6,1	1387	6,1	4,7	-	7,9
	Castelló	4,3	3,8	1,8	-	8,4	768	9,3	6,7	-	12,8
	València	2,7	3,1	1,9	-	5,4	1687	4,6	3,4	-	6,1
Extremadura	Badajoz	3,6	3,8	2,3	-	6,6	1344	6,5	5,2	-	8,2
	Cáceres	4,1	4,9	2,8	-	8,9	1031	10,4	7,4	-	14,6
Galicia	Coruña, A	2,1	2,5	1,4	-	4,9	960	3,8	2,4	-	6
	Lugo	3,4	3,3	1,3	-	9,1	642	4,3	2,6	-	7
	Ourense	5,0	4,1	2,2	-	9,8	450	6,2	4,1	-	9,1
	Pontevedra	3,1	3,0	2,0	-	5,1	1051	4,8	3,5	-	6,6
Madrid	Madrid	13,1	14,9	12,4	-	17,9	2734	18,6	16,7	-	20,6
Murcia	Murcia	2,6	1,7	0,9	-	4,2	1389	6,1	4,3	-	8,6
Navarra	Navarra	7,2	7,2	5,2	-	10,1	1422	14,3	11,7	-	17,2
Euskadi	Araba/Álava	8,7	8,9	6,1	-	13,6	616	12	9,5	-	15
	Gipuzkoa	3,4	3,3	1,6	-	6,6	866	6,4	4,7	-	8,6
	Bizkaia	5,1	4,7	2,9	-	8,2	1146	8,4	6,3	-	11
Rioja, La	Rioja, La	4,2	4,9	3,3	-	7,6	1127	8,2	6,3	-	10,5

Table ST1. Distribution of SARS-CoV-2 seroprevalence in Spain: Comparison of Rounds 1-3 and Rounds 1-4 results.

		Seroconversion				
		Rounds 1,2,3 –>4				
CC.AA.	Province	Number	%	CI (95%)		
Andalucía	Almería	624	2,4	1,2	-	4,5
	Cádiz	928	2,9	1,7	-	4,9
	Córdoba	825	2,1	1,2	-	3,4
	Granada	774	5,1	3,3	-	7,8
	Huelva	778	2,7	1,3	-	5,6
	Jaén	738	3,6	2,3	-	5,4
	Málaga	952	2,6	1,5	-	4,4
	Sevilla	1326	5,3	3,1	-	9
Aragón	Huesca	526	4	2,1	-	7,6
	Teruel	483	7	5,2	-	9,4
	Zaragoza	954	6,6	4,5	-	9,6
Asturias	Asturias	1500	4	3	-	5,3
Balears, Illes	Balears	730	2,3	1,3	-	3,9
Cantabria	Cantabria	1261	2,4	1,6	-	3,7
Castilla y León	Ávila	487	5,3	2,4	-	11,3
	Burgos	618	5,1	2,7	-	9,4
	León	703	2	1	-	3,9
	Palencia	568	5	3	-	8,3
	Salamanca	614	4,2	2,8	-	6,5
	Segovia	458	4,6	2,5	-	8,5
	Soria	525	2,9	1,2	-	6,5
	Valladolid	776	3	1,9	-	4,6
	Zamora	509	6,2	4,1	-	9,2
Castilla-La Mancha	Albacete	745	4,3	2	-	9,1
	Ciudad Real	789	3,8	2,1	-	6,9
	Cuenca	536	3,5	1,5	-	7,9
	Guadalajara	513	4,9	2,4	-	9,9
	Toledo	680	3,7	2	-	6,9
Catalunya	Barcelona	2472	4,1	3	-	5,5
	Girona	722	7	4,4	-	11,1
	Lleida	510	8,2	4,3	-	15
	Tarragona	606	3,8	1,9	-	7,4
C. Valenciana	Alacant	1226	2,2	1,3	-	3,8
	Castelló	709	5	3,3	-	7,7
	València	1540	1,9	1,1	-	3
Extremadura	Badajoz	1241	2,9	1,9	-	4,4
	Cáceres	932	6,3	3,9	-	10
Galicia	Coruña, A	849	1,7	0,7	-	4,2
	Lugo	558	0,9	0,3	-	2,8
	Ourense	401	1,2	0,4	-	3,6
	Pontevedra	889	1,7	0,8	-	3,3
Madrid	Madrid	2113	5,5	4,2	-	7,2
Murcia	Murcia	1130	3,5	2,2	-	5,5
Navarra	Navarra	1261	7,1	5,3	-	9,4
Euskadi	Araba/Álava	512	3,3	1,6	-	6,4
	Gipuzkoa	773	3	1,8	-	5,2
	Bizkaia	970	3,3	2	-	5,5
Rioja, La	Rioja, La	1007	4	2,6	-	6,2

Table ST2. Estimation of seroconversion incidence of anti-SARS-CoV-2 antibodies: Comparison of negative to positive status from Rounds 1-3 to Round 4.

S3 Estimation COVID-19 detection and naive population in the Spanish population in March 2021

In a previous section, S2, we have explained the existence and importance of a seroprevalence study in a subpopulation, for each province and during a certain period of time (between different waves and in the middle of one of them). The level of presence of the SARS-CoV-2 virus (or its antibodies) in the total population is a very important indicator to ensure that the evolution of the virus in our study is not influenced by this, but mainly by the mobility of the population and weather conditions. If the proportion of the population with the virus (or its antibodies) was, during the period September 2020 - March 2021, too high, it would imply that the evolution of the virus is limited due to a lack of susceptible individuals for the free circulation of the virus. This limitation is not unique according to the level of seroprevalence, but the correlation that we are trying to demonstrate in this article could be altered due to the emergence of new virus variants (see section S4) or the level of vaccination (as discussed previously in S1).

In Spain, in addition to the aforementioned ENE-COVID study, we have the official data on the number of daily cases per province from the *Instituto de Salud Carlos III* (ISCIII) (see Fig SF2 [1]. Obviously, these daily cases are reported to institutions based on visits to primary care centers (CAP) and hospital admissions. This implies that these people had symptoms (mild or severe) prompting them to seek medical attention. These data clearly do not reflect all the people who contracted the virus (whether they were asymptomatic or simply did not go to a medical center). On the other hand, due to its constant monitoring design in a subgroup of the population, the ENE-COVID study does allow for the detection of all these positive people who are not detected by medical centers. It can be expected, then, that the cases reported in the official data by the health personnel will underestimate the real circulation data of the virus.

To demonstrate that the percentage of seroprevalence in the population of each province is not large enough to influence the correlation of our study, we have chosen the most unfavorable scenario for us. So, in order to more accurately evaluate the total accumulated number of cases (or at least consider the highest number of cases possible from real data), we have combined both

[1] Ref https://cnecovid.isciii.es/covid19/

studies, the official daily cases reported by the ISCIII and the epidemiological study ENE-COVID. The idea is simple: between rounds 3 (08/06–22/06/2020) and 4 (16/11–29/11/2020) of the study, Spain is experiencing the second wave's ascendance, with a very notable increase in the number of cases in all provinces, especially in August and September. Comparing cumulative cases between 22/06/2020 and 29/11/2020 between official data and ENE-COVID data, we can obtain a proportion of positive cases relative to the total population of each study (the total population of each province for the ISCIII study and the total subpopulation of each province for the ENE-COVID study). As similar case peaks occur in the subsequent months (December 2020 - March 2021), see SF2, we will apply these same proportions (by province) to the period 29/11/2020 (Round 4, and last ENE-COVID Round) - 04/03/2021 (end of our study). This allows us to estimate the population percentage that had encountered the SARS-CoV-2 virus by March 2021. Figure SF3 and Table ST3 show the results calculated from the ENE-COVID data, which are always higher than the official data reported by healthcare workers, as is logical and had been predicted in earlier paragraphs.

The average cumulative case percentage (people who have been in contact with the virus at some point) for the entire national territory as of March 4, 2021, is 5.6% according to ISCIII data and 13.1% (CI 10.6-16.4%) according to our estimates.

The most extreme cases are located in the central *Comunidades Autónomas* (CC.AA.) of Madrid and Castilla-La Mancha, followed by Castilla y León and Aragón (central-north, their territory borders the first two) and by Catalunya and Navarra (in northeastern and northwestern directions from Aragón, respectively). All of them exceed the average of Spain. On the other hand, the northwest autonomous communities (Galicia, Asturias, and Cantabria) show the lowest percentage by far, with an average of less than $<7.5\%$.

For our study, although, some provinces have a high estimated percentage ($\geq 20\%$), cases of Madrid and most of Castilla-La Mancha, the rest of the provinces are close to or below 15%, so we prove the point done in the main manuscript: population immunity or lack of susceptible population are not the main drivers of the epidemics.

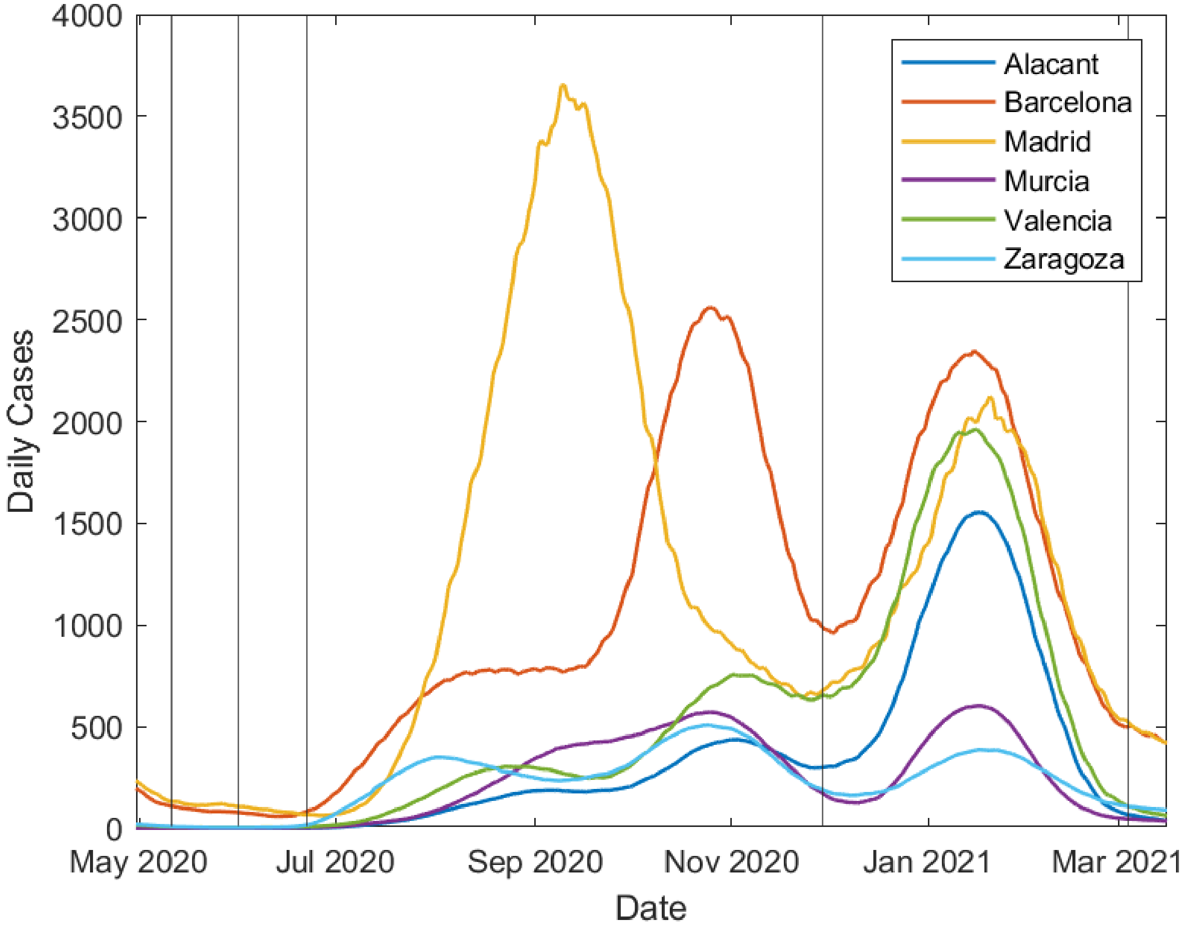

Figure SF2. Smoothed representation of the daily number of COVID-19 cases reported in Spanish provinces most affected by the pandemic between May 2020 and March 2021 (with cumulative cases exceeding 70,000). To simplify the figure, not all 48 provinces are included. Vertical lines show the dates of the four different ENE-COVID Rounds and the final date for this study.

			ISCIII Cases 04/03/2021		ENE-Covid Expected 04/03/2021		
CC.AA.	Province	Total Population (2021)	N	%	N	%	CI (95%)
Andalucía	Almería	731792	36036	4,92	71785	9,8	7,6 - 12,8
	Cádiz	1245960	50398	4,04	124422	10,0	8,5 - 12,0
	Córdoba	776789	25061	3,23	49585	6,4	5,1 - 8,1
	Granada	921338	60035	6,52	113439	12,3	9,9 - 15,5
	Huelva	525835	17339	3,30	43988	8,4	6,4 - 11,8
	Jaén	627190	26074	4,16	66728	10,6	8,2 - 13,9
	Málaga	1695651	51281	3,02	155781	9,2	7,2 - 12,2
	Sevilla	1947852	65459	3,36	228525	11,7	9,4 - 14,7
Aragón	Huesca	224264	16100	7,18	22137	9,9	7,5 - 13,1
	Teruel	134545	12981	9,65	22079	16,4	13,8 - 19,7
	Zaragoza	967452	76438	7,90	154115	15,9	13,3 - 19,0
Asturias	Asturias	1011792	42269	4,18	97467	9,6	8,2 - 11,2
Balears, Illes	Balears	1173008	48143	4,10	103898	8,9	7,4 - 10,8
Cantabria	Cantabria	584507	24558	4,20	46061	7,9	6,4 - 10,0
Castilla y León	Ávila	158421	6911	4,36	26087	16,5	13,0 - 20,8
	Burgos	356055	20612	5,79	46340	13,0	9,4 - 17,7
	León	451706	30701	6,80	51076	11,3	8,0 - 15,9
	Palencia	159123	11135	7,00	25585	16,1	12,8 - 20,2
	Salamanca	327338	22046	6,73	53178	16,2	13,2 - 19,9
	Segovia	153663	11134	7,25	32272	21,0	17,2 - 25,7
	Soria	88747	5897	6,64	18207	20,5	16,6 - 25,1
	Valladolid	519361	31571	6,08	62566	12,0	9,0 - 15,9
	Zamora	168725	10088	5,98	28440	16,9	13,4 - 21,4
Castilla-La Mancha	Albacete	386464	23106	5,98	85482	22,1	18,3 - 26,8
	Ciudad Real	492591	33370	6,77	94692	19,2	15,8 - 23,5
	Cuenca	195516	14190	7,26	43163	22,1	18,4 - 26,5
	Guadalajara	265588	17164	6,46	56879	21,4	17,4 - 26,3
	Toledo	709403	52225	7,36	129914	18,3	14,7 - 22,6
Catalunya	Barcelona	5714730	364462	6,38	893473	15,6	13,4 - 18,2
	Girona	786596	49161	6,25	132736	16,9	13,9 - 20,9
	Lleida	439727	32277	7,34	74405	16,9	12,4 - 23,4
	Tarragona	822309	43027	5,23	71760	8,7	6,7 - 11,8
C. Valenciana	Alacant	1881762	109879	5,84	210952	11,2	9,8 - 13,0
	Castelló	587064	29465	5,02	112923	19,2	16,6 - 22,7
	València	2589312	166553	6,43	211666	8,2	7,0 - 9,7
Extremadura	Badajoz	669943	28111	4,20	60946	9,1	7,8 - 10,8
	Cáceres	389558	17822	4,57	65606	16,8	13,8 - 21,0
Galicia	Coruña, A	1120134	45538	4,07	70445	6,3	4,9 - 8,5
	Lugo	326013	11011	3,38	16816	5,2	3,5 - 7,9
	Ourense	305223	13007	4,26	22797	7,5	5,4 - 10,4
	Pontevedra	944275	34151	3,62	66614	7,1	5,8 - 8,9
Madrid	Madrid	6751251	432103	6,40	1449283	21,5	19,6 - 23,5
Murcia	Murcia	1518486	76394	5,03	124379	8,2	6,4 - 10,7
Navarra	Navarra	661537	44665	6,75	106347	16,1	13,5 - 19,0
Euskadi	Araba/Álava	333626	16535	4,96	44793	13,4	10,9 - 16,4
	Gipuzkoa	726033	39787	5,48	52714	7,3	5,6 - 9,5
	Bizkaia	1154334	60609	5,25	112193	9,7	7,6 - 12,3
Rioja, La	Rioja, La	319796	26028	8,14	36401	11,4	9,5 - 13,7

Table ST3. Comparison of number and percentage of the seroprevalence of COVID-19 for Spanish provinces at March 4th, 2021. *Instituto de Salud Carlos III* (ISCIII) data is the national official cumulative cases and ENE-Covid numbers are the ones estimated from the study of a subpopulation group during several weeks. The method is explained in section S3.

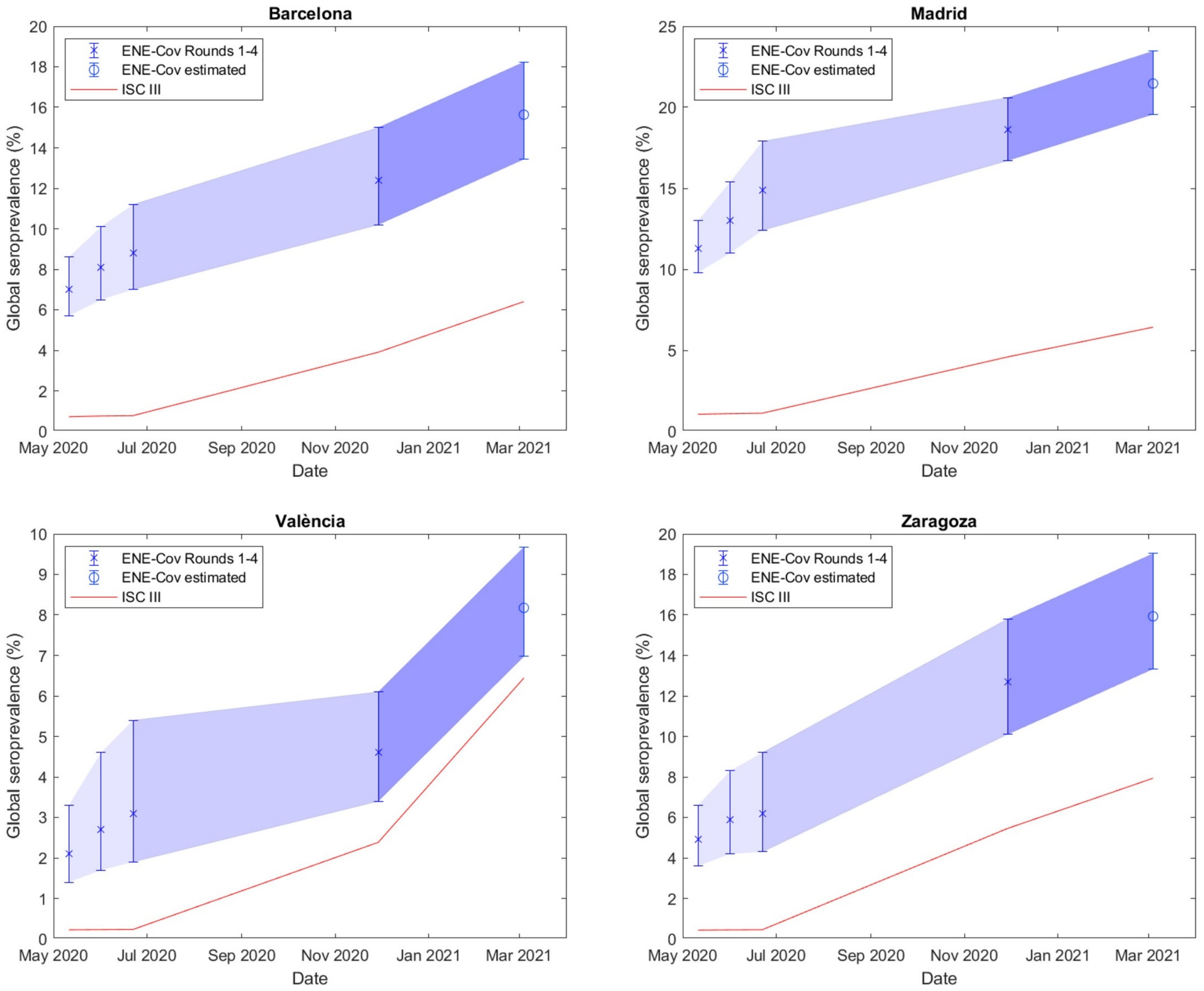

Figure SF3. Comparison of the percentage of the population with seroprevalence of COVID-19 (detected by ENE-COVID study) and the percentage of cumulative cases reported by ISCIII data set in four high-impact Spanish provinces (Barcelona, Madrid, Valencia and Zaragoza) between May 2020 and March 2021. These provinces were selected for their high number of cumulative cases and for representing different regions of Spain.

S4 The emergence of the Alpha variant in Spain in early 2021

In the main article, we aim to investigate a period where the rate of epidemiological spread is not mainly influenced by factors such as the number of susceptible individuals, which is largely influenced by prior infections, the circulating variant, and the decrease in population immunity. The impact of prior infections, those infected during the second wave, and those with decreased immunity have been discussed in the previous section. In this section, we will analyze the impact of the change in the variant on the second and early stages of the third wave in Spain from December 2020 to March 2021.

The second wave was largely triggered by the relaxation of non-pharmacological containment measures after the summer of 2020 and was dominated by the Alpha variant (or B.1.177 variant). Similarly, the third wave in Spain, which closely followed the second wave, was a direct result of relaxed measures implemented during the holiday season. Additionally, a highly transmissible variant B.1.1.7, made a strong appearance. This variant was first detected in the UK in December and was later found in Spain in limited numbers at the end of the same month. However, in the following weeks, it became the predominant variant in all regions of the country.

The arrival and spread of the new variant, which replaced the previous B.1.177 variant, may have unintended consequences for our study as it changes the paradigm from the previous months. Figure SF4 displays the percentage of the new variant in different *Comunidades Autónomas* (CC.AA.) based on official data from the *Ministerio de Sanidad* (Department of Health)[4].

The CC.AA. of Galicia, Asturias, and Cantabria (which are all located together in the northwest and north, respectively) showed the highest percentages of the Alpha variant in the first weeks of its appearance. Around the second week of January, all three regions exceeded 20% of this variant compared to the rest of the sequenced variants. Galicia particularly stood out for its high initial percentage of the variant, which may be due to the small number of samples taken - only between 50 and 60 samples were taken during the third and fourth weeks, respectively. As we approached week 12 (the last shown in Figure SF4 and the last week of March 2021), the number of analyzed samples from Galicia increased to around 250 per week, bringing the curve closer to the behavior

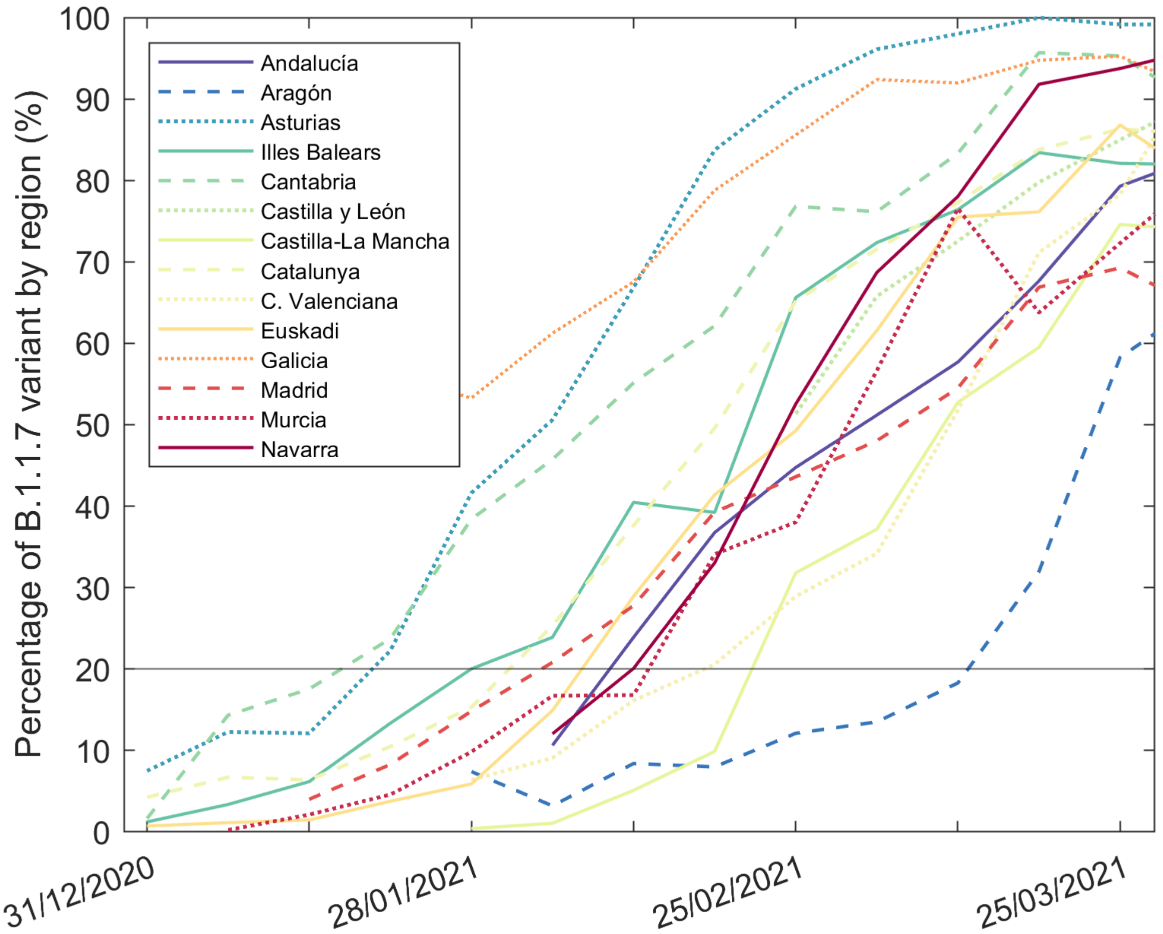

Figure SF4. Evolution of B.1.1.7 (Alpha) variant in different CC.AA. during early weeks of 2021.

seen in the rest of the Spanish territory [2].

The remaining regions exceeded the 20% threshold during February (except Aragón, which took a few more weeks) and showed a very similar rapid increase, reaching between 70% and 90% of the total sequenced samples as the Alpha variant by early March. Given this information, we have re-analyzed the mobility and meteorological data, ending the analysis in January for Galicia, Asturias, and Cantabria and ending the analysis in February for the rest of the Autonomous Communities. However, we found no significant differences in any of our analyses.

[2]Data taken from updates on the epidemiological situation of the SARS-CoV-2 variants published intermittently on the *Ministerio de Sanidad* website.

S5 SIR model to exemplify time-lags between external signals and case-count growth rate

We advance here a simple model to test how a SIR structure can provide a good example of how causal relations between non-pharmacological interventions or meteorological variables lead to outcomes in the growth rate with delays between changes in the external signal and effects seen in the growth rate. For this, we will use a SIR toy model where the key driver is not only the level of the susceptible population but how the ability to infect depends smoothly on an external signal that we call ES. This model helps us explain how a well-defined model where the external signal causes increases in the growth rate of the epidemics is reflected in the correlation peak at a certain time delay between the external signal driving the infection and the growth rate.

Consider the SIR model

$$\frac{dS}{dt} = -\alpha(t)SI - n_o\eta \quad \frac{dI}{dt} = \alpha(t)SI - \beta I + n_o\eta \quad \frac{dR}{dt} = \beta I \quad N_w = \alpha(t)SI + n_o\eta \tag{1}$$

with $\Delta t = 1$ day, η random number between zero and one, and initial conditions $S_o = Np = 10^6$, $I_o = 10$ (for a typical Spanish province in August 2020), $R_o = 0$, and $\beta = 1/5$ days^{-1}. The key point of the model is to provide a relation between α and a given external signal and check how the growth correlates with the signal that determines α. In this model, the external signal, be it a mobility reduction, the average temperature, or any other, causally affects the infection rate.

In order to be able to have growth rates that abate with a rather low number of infected before the susceptible population drops significatively, we consider that α has a basal rate and that it can increase when external signal changes, being the maximum α constrained using a tanh relation between signal and thus infection rate.

$$\alpha = \frac{1}{Np}(\alpha_o - \Delta\alpha \tanh[k_s(ES(t) - ES_o)]). \tag{2}$$

The saturation in the infection rate α comes naturally from the fact that geography forbids a full perfect mixing of the population. People in one city are not permanently in contact with others, and the infection rate must necessarily saturate.

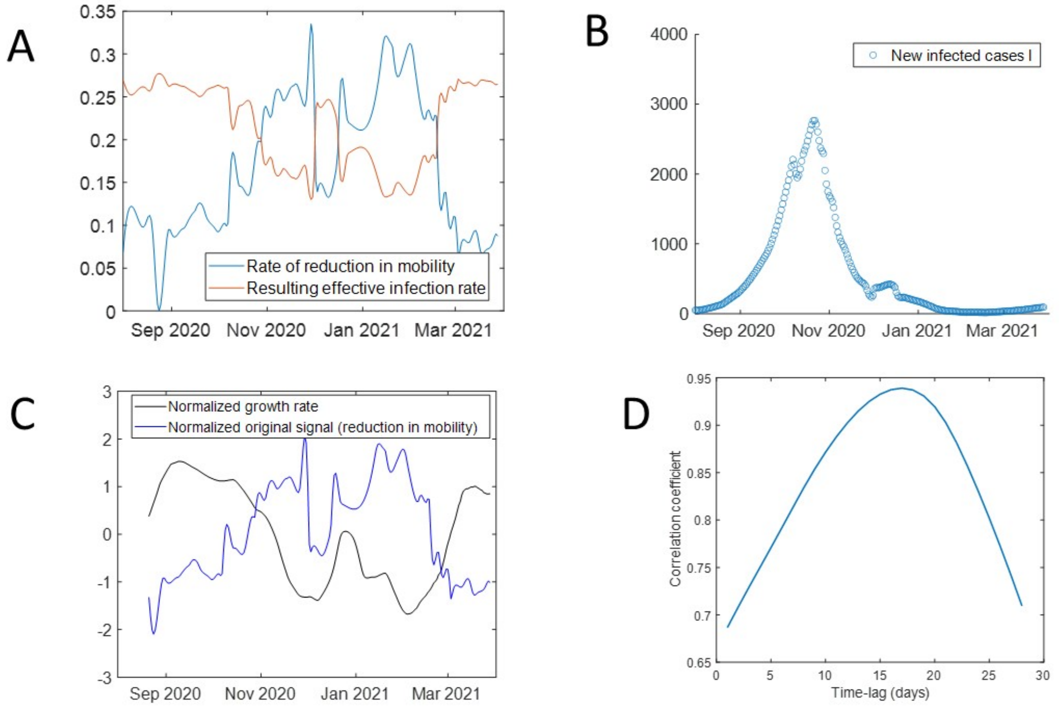

Figure SF5. Panel A: Rate of reduction of mobility as an external signal used in the model together with the resulting αN_p obtained from this signal. Panel B. New cases as obtained in the SIR model from the previous external signal Panel C: The external signal is shown without average and normalized standard deviation together with the growth rate, computed as in the manuscript, also normalized. Panel D: the resulting correlation coefficient between both signals is computed depending on the time lag established between both. Maximum correlation is obtained when the external signal is correlated with a shift of 18 days.

To implement the model we chose a typical external signal which is initially low (as in low reduction in mobility), then increases (high reduction in mobility, leading to higher mobility), and then finally decreases again. In this framework, α is high at the beginning, allowing for epidemic growth, then it drops, suggesting the end of the epidemic wave before reaching all susceptible possible. The A panel of the figure SF5 shows the external signal we have used for this toy model as a typical one in the manuscript (ES would be the reduction in mobility) and the associated αN_p constructed from this signal with $k_s = 0.1$, $\alpha_o = 0.2$, $\Delta\alpha = 0.08$ and $ES_o = 0.2$. Panel B shows the corresponding number of infected I showing a clear wave-like epidemic. The third panel shows the associated growth rate as computed in the main manuscript in direct comparison with the original signal. Both signals are here normalized. We can clearly observe how there is a delay between the external signal and the growth rate. We compute in panel D the correlation between both signals using different time-delays showing that it peaks at around 18 days.

It is clear that different signals and different parameters of the model can lead to different changes in the peak between this correlation. The purpose here is not to make a detailed analysis of all the possibilities but to show how a deterministic model produces a naturally large correlation coefficient, provided there is a time-delay, between an external signal which has a direct effect on the infection rate and growth rate. We also find that they are in the ballpark of those found in the main manuscript.

S6 Figure of the incidence of COVID19 and its growth rate in Spanish provinces from 4th of September 2020 to 4th of March 2021

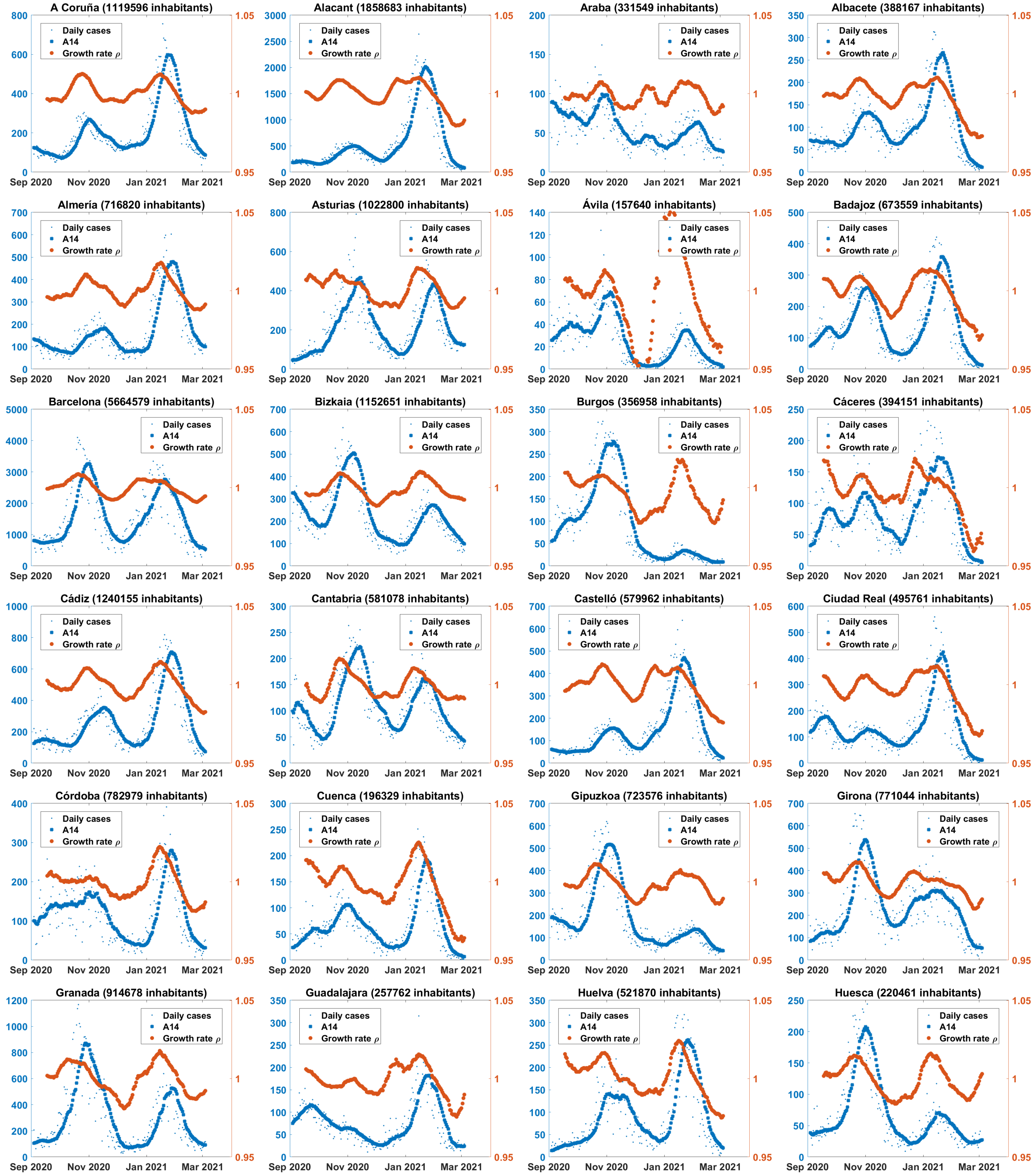

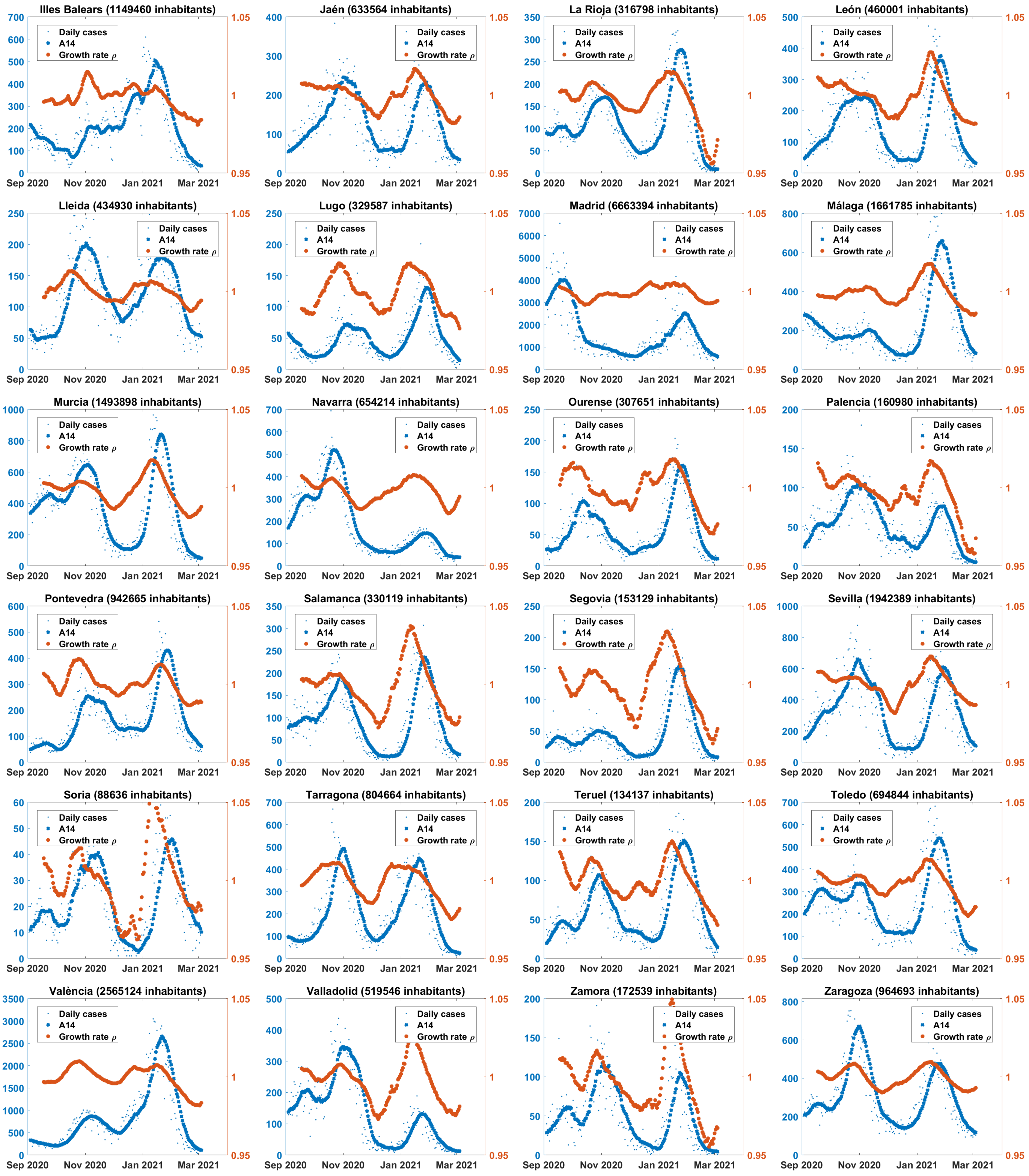

Figure SF6. Incidence of COVID19 and its growth rate in Spanish provinces from 4th of September 2020 to 4th of March 2021.

S7 Figure of the evolution of the reduction in mobility from Facebook and MITMA data sources.

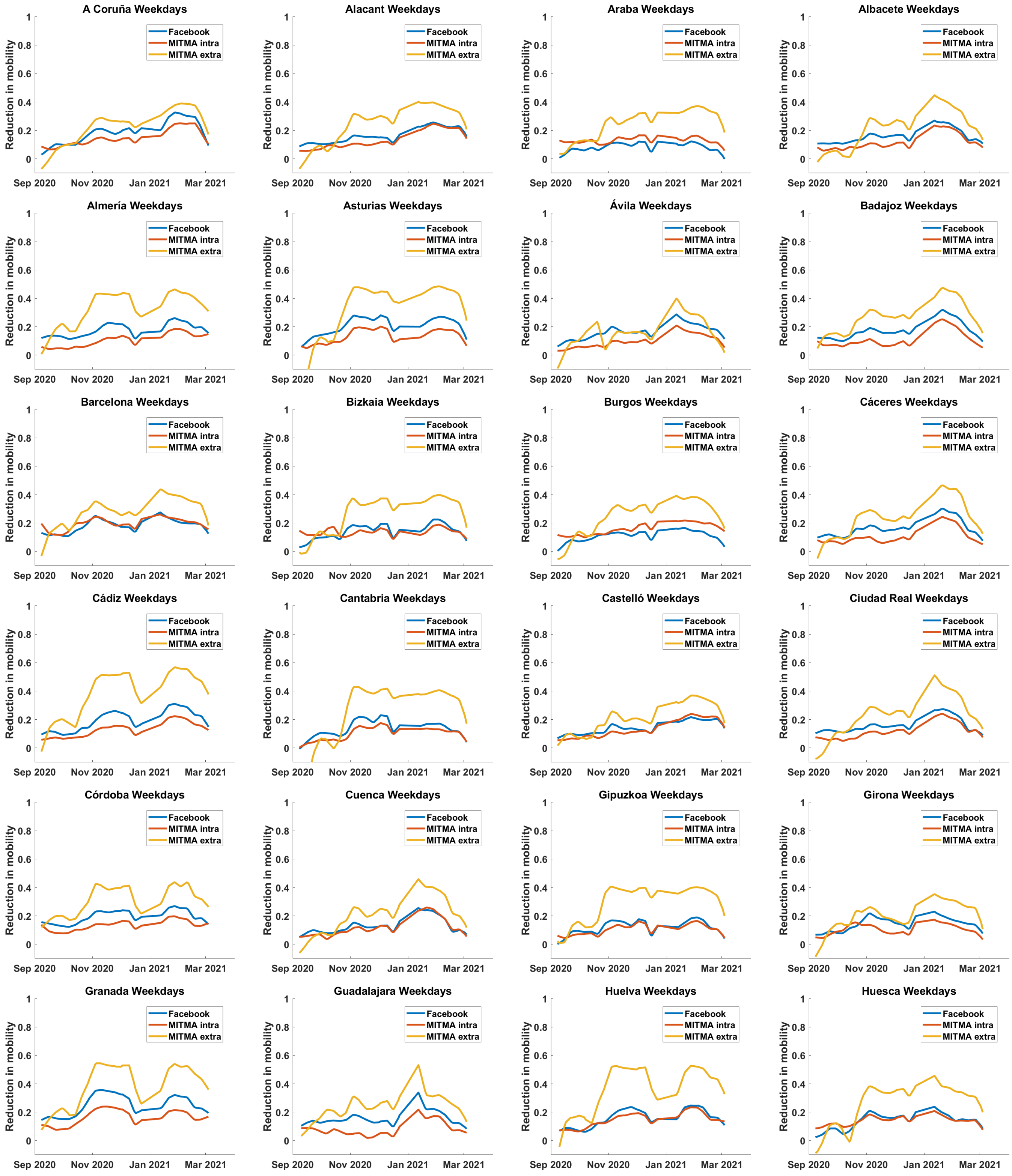

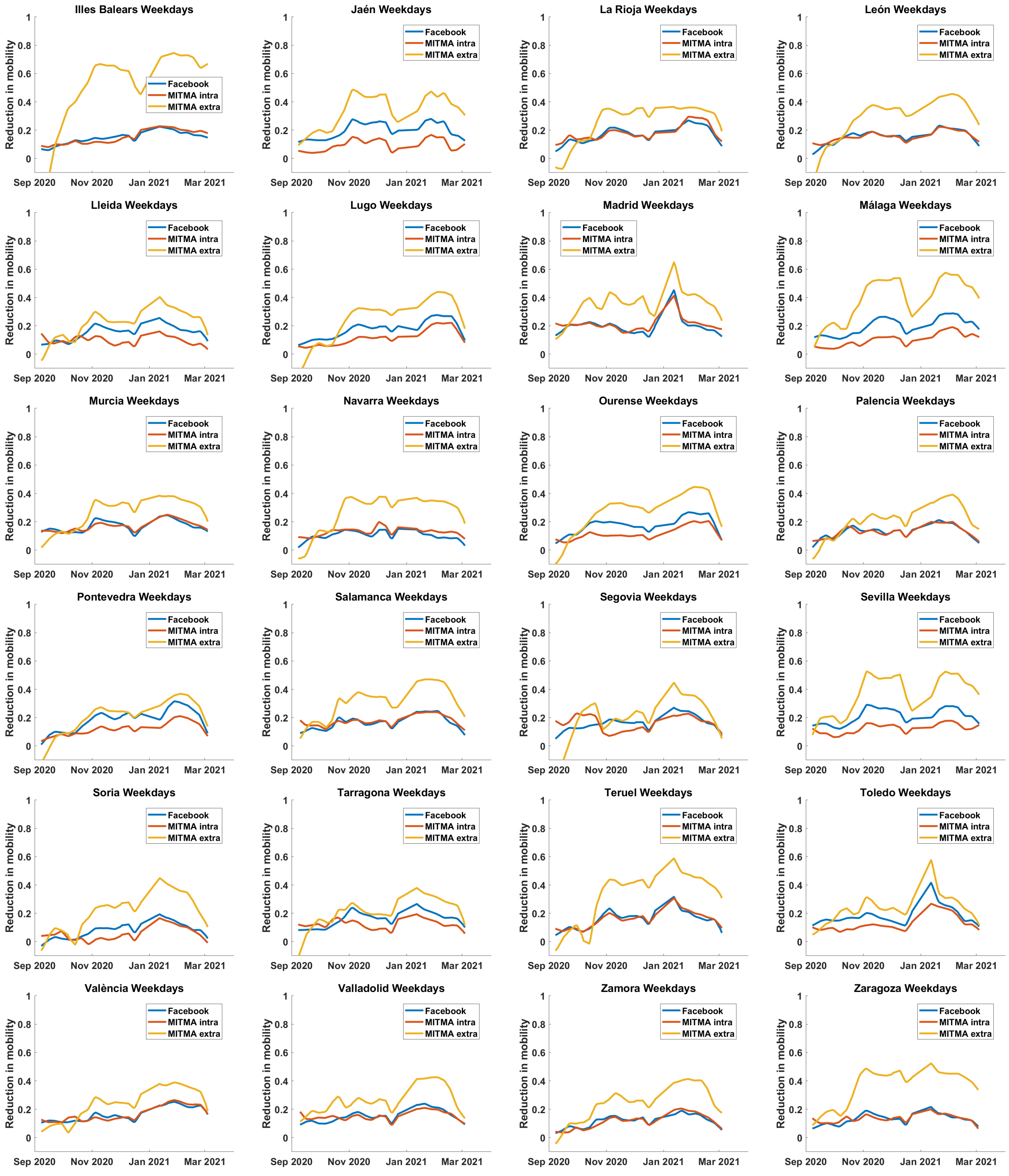
Illes Balears Weekdays
Jaén Weekdays
La Rioja Weekdays
León Weekdays
Lleida Weekdays
Lugo Weekdays
Madrid Weekdays
Málaga Weekdays
Murcia Weekdays
Navarra Weekdays
Ourense Weekdays
Palencia Weekdays
Pontevedra Weekdays
Salamanca Weekdays
Segovia Weekdays
Sevilla Weekdays
Soria Weekdays
Tarragona Weekdays
Teruel Weekdays
Toledo Weekdays
València Weekdays
Valladolid Weekdays
Zamora Weekdays
Zaragoza Weekdays
Facebook
MITMA intra
MITMA extra
Reduction in mobility
0
0.2
0.4
0.6
0.8
1
Sep 2020
Nov 2020
Jan 2021
Mar 2021

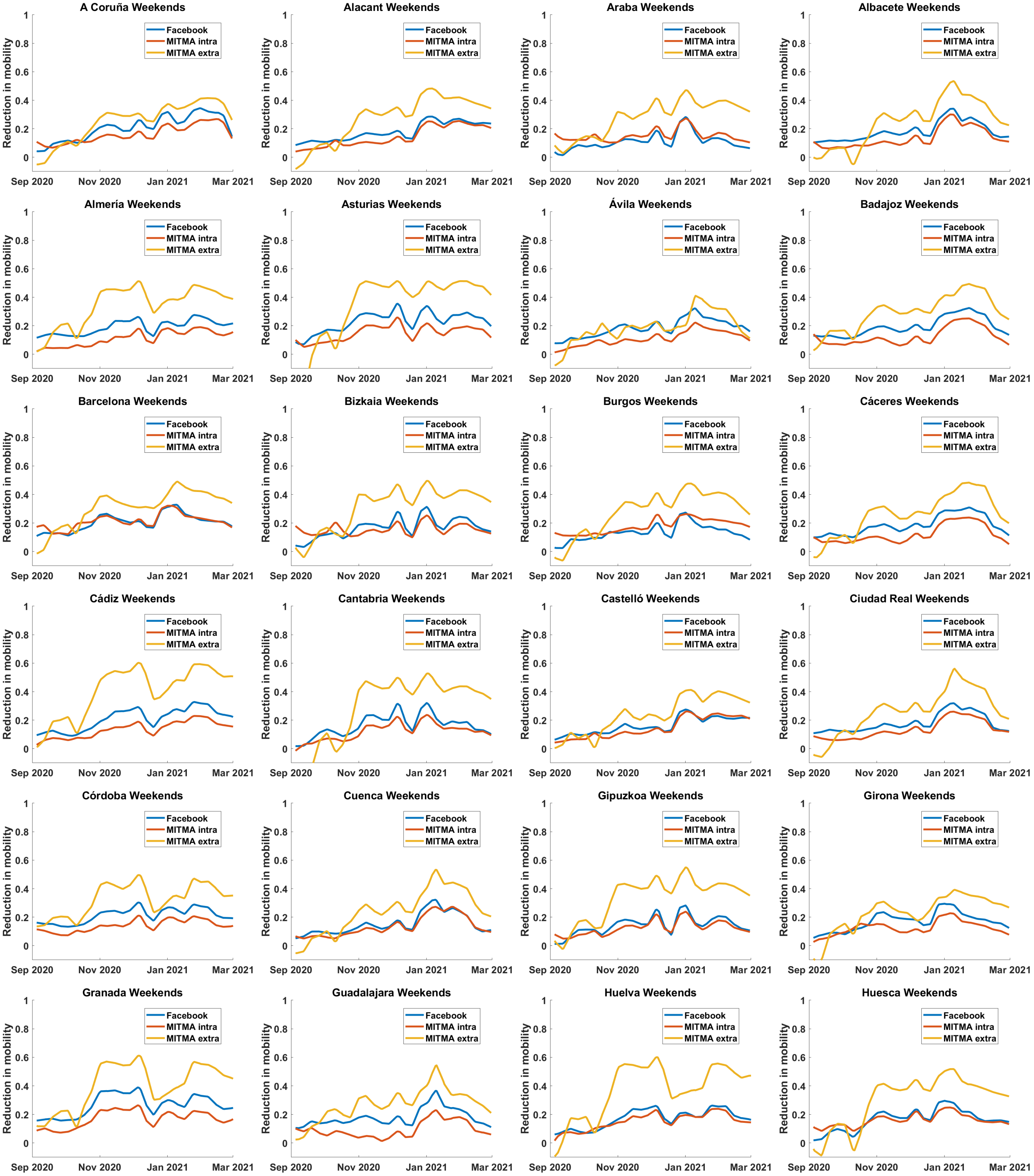
A Coruña Weekends
Alacant Weekends
Araba Weekends
Albacete Weekends
Almería Weekends
Asturias Weekends
Ávila Weekends
Badajoz Weekends
Barcelona Weekends
Bizkaia Weekends
Burgos Weekends
Cáceres Weekends
Cádiz Weekends
Cantabria Weekends
Castelló Weekends
Ciudad Real Weekends
Córdoba Weekends
Cuenca Weekends
Gipuzkoa Weekends
Girona Weekends
Granada Weekends
Guadalajara Weekends
Huelva Weekends
Huesca Weekends
Facebook
MITMA intra
MITMA extra
Reduction in mobility
Sep 2020
Nov 2020
Jan 2021
Mar 2021

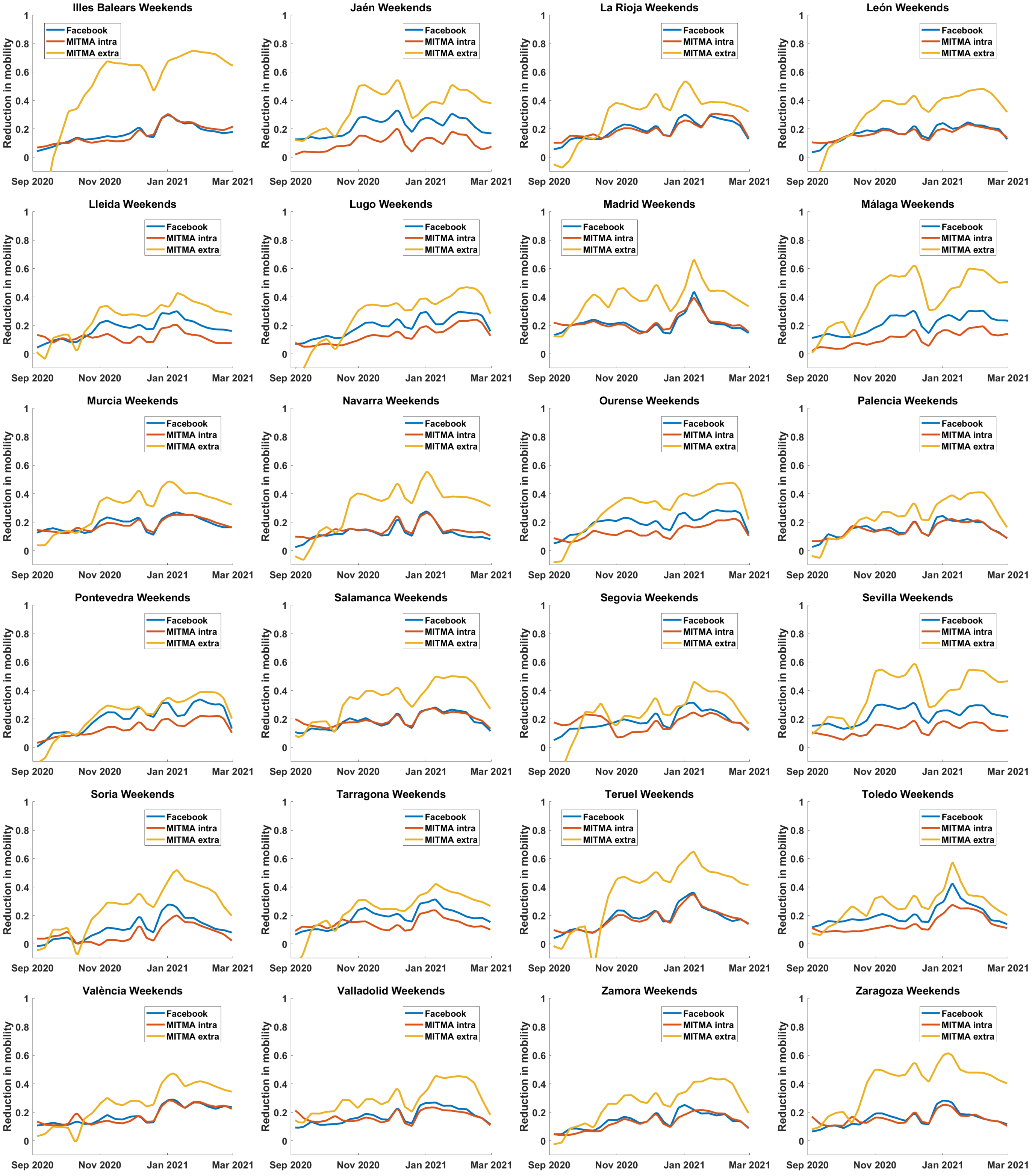

Figure SF7. Evolution of the reduction in mobility obtained by processing Facebook and MITMA data sources.

S8 Figure of the evolution of temperature, dew point and UV radiation from the Copernicus Climate Data Store

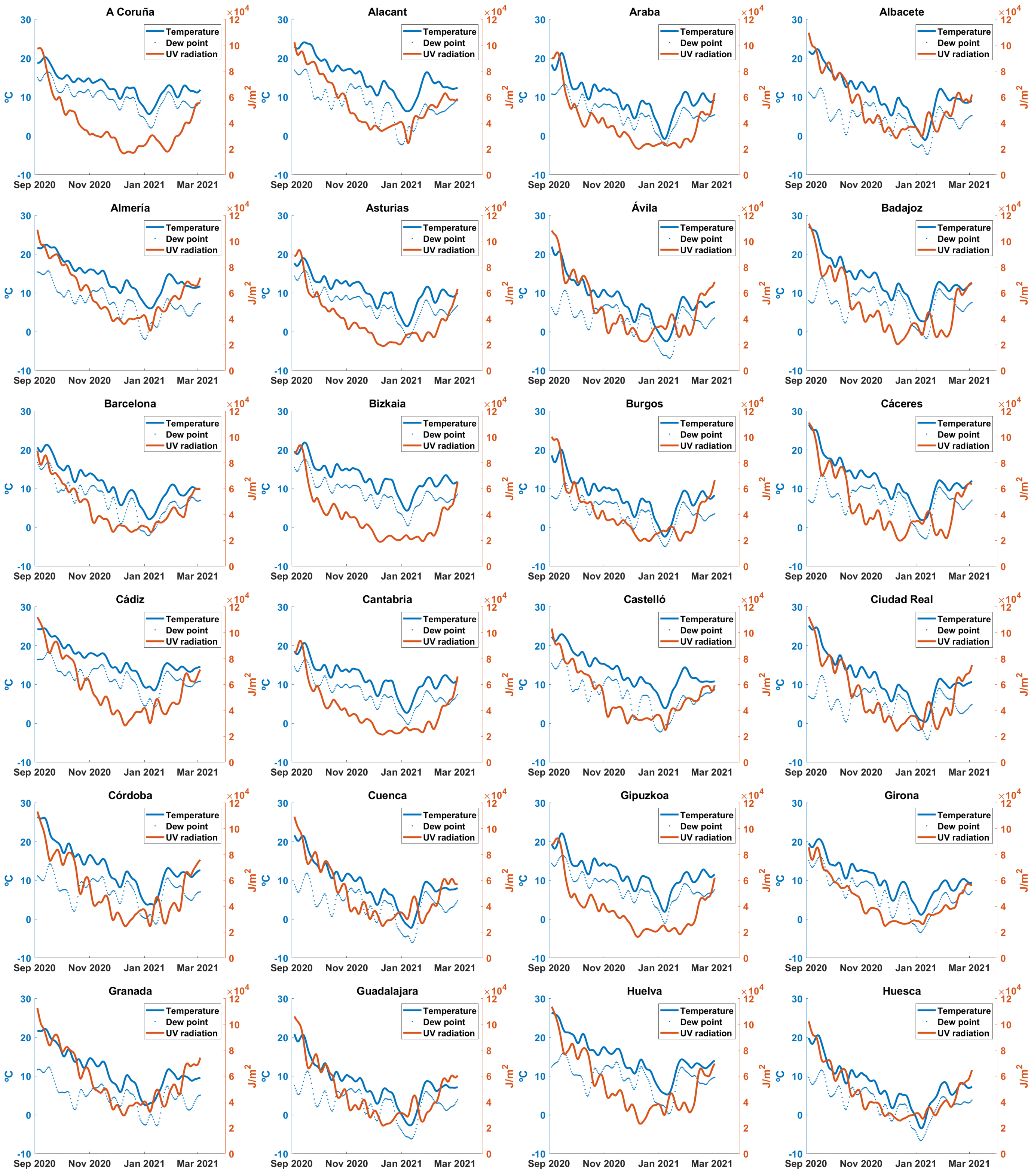

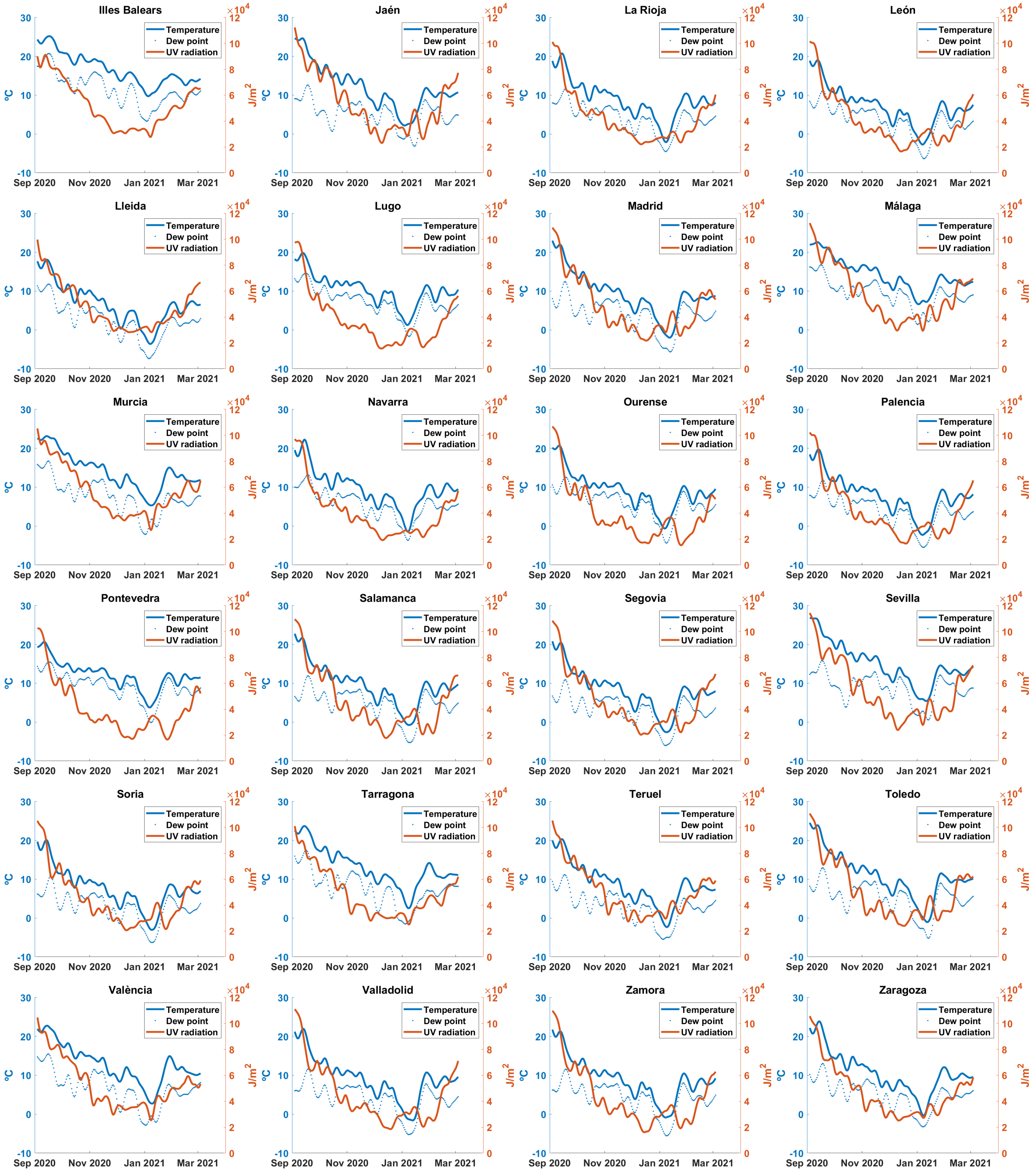

Figure SF8. Evolution of temperature, dew point and UV radiation processed signals from the Copernicus Climate Data Store for all Spanish provinces.

References

1. Polack, F. P. *et al.* *New Engl. J. Medicine* **383**, 2603–2615, DOI: 10.1056/NEJMoa2034577 (2020). PMID: 33301246, https://doi.org/10.1056/NEJMoa2034577.

2. Instituto de Salud Carlos III. Estudio ENE-COVID: Informe final (2020 [Online]).

3. Pollán, M. *et al.* Prevalence of sars-cov-2 in spain (ene-covid): A nationwide, population-based seroepidemiological study. *The Lancet* **396**, 535–544, DOI: 10.1016/s0140-6736(20)31483-5 (2020).

4. Ministerio de Sanidad. Actualización de la situación epidemiológica de las variantes de SARS-CoV-2 de importancia en salud pública en España (2021 [Online]).
